# Centrin-Deleted *Leishmania donovani* Parasites Help CD4^+^ T Cells to Acquire Th1 Phenotype and Multi-Functionality Through Downregulation of CD200–CD200R Immune Inhibitory Axis

**DOI:** 10.3389/fimmu.2018.01176

**Published:** 2018-06-04

**Authors:** Rakesh K. Singh, Sreenivas Gannavaram, Nevien Ismail, Amit Kaul, Mallikarjuna Rao Gedda, Hira L. Nakhasi

**Affiliations:** ^1^Division of Emerging and Transfusion Transmitted Diseases, Center for Biologics Evaluation and Research, United States Food and Drug Administration, Silver Spring, MD, United States; ^2^Department of Biochemistry, Institute of Science, Banaras Hindu University, Varanasi, India; ^3^Johns Hopkins Medical Institution, Johns Hopkins University, Baltimore, MD, United States

**Keywords:** CD-200, CD200-R, live attenuated vaccines, *Leishmania*, vaccine immunity, multi-functionality

## Abstract

The protozoan parasite *Leishmania* has evolved several strategies to undermine host defense mechanisms by inducing Th2-type adaptive immunity and suppressing effector functions of Th1 phenotype. In our earlier studies, using centrin gene-deleted *Leishmania* (LdCen^−/−^) parasites as an immunogen, we have shown induction of an effective Th1-type immunity and robust memory responses that mediate protection against virulent challenge. However, role of inhibitory signals in *Leishmania* vaccine induced immunity in general, and LdCen^−/−^ in particular has not been studied. Herein, we report that immunization with LdCen^−/−^ parasites produces more functional Th1-type CD4^+^ T cells *via* downregulation of CD200–CD200R immune inhibitory axis compared to wild-type infection. We found that expression of CD200 and CD200R was significantly reduced in LdCen^−/−^ infection compared to wild-type infection. Diminished CD200–CD200R signaling in LdCen^−/−^ infection enabled proliferation of CD4^+^ T cells and resulted in the induction of pro-inflammatory cytokines and suppression of anti-inflammatory response. The effects of diminished CD200–CD200R signaling by LdCen^−/−^ were most evident in the suppression of IL-10-producing CD4^+^ T cells that helped enhance more Th1 cytokine producing and multi-functional T cells compared to wild-type infection. *In vivo* blocking of CD200 expression with anti-CD200 treatment in wild-type infected mice limited Th2 response as indicated by reduction of IL-10-producing Tr1 cells and reduced parasite burden. On the other hand, treatment with anti-CD200 improved the LdCen^−/−^ vaccine-induced multifunctional response and reduction in splenic parasite load upon challenge. Taken together, these studies demonstrate the role of CD200–CD200R signals in the protection induced by LdCen^−/−^ parasites.

## Importance

Our previous studies have shown that immunization with centrin gene-deleted live attenuated *Leishmania* (LdCen^−/−^) parasites enables induction of a strong protective immunity. However, the immune mechanisms, especially early interaction between antigen-presenting cells and the naïve T cells that promote the establishment of protective immunity in the immunized host, are not well understood. This study demonstrates that immunization with live attenuated LdCen^−/−^ parasites results in limited but specific activation of CD200–CD200R immune inhibitory axis and facilitates the induction of pro-inflammatory cytokines and suppression of anti-inflammatory response. In contrast, infection with virulent wild-type *Leishmania donovani* parasites resulted in a strong induction of CD200–CD200R immune inhibitory signals in both DCs and the CD4^+^ T cells. Additionally, we found that subdued induction of CD200–CD200R signaling by LdCen^−/−^ parasites is important in the acquisition of a protective multifunctional phenotype by the CD4^+^ T cells following immunization. We also show that antigen-experienced CD4^+^ T cells expressing CD200R receptor are a major IL-10-producing phenotype. This is significant as the *Leishmania* parasite is known to evade host defense mechanisms by inducing Th2-type adaptive immunity and suppressing effector functions of Th1 phenotype.

This study demonstrates the attenuation properties of live attenuated vaccines in their role in diminishing CD200–CD200R signaling besides other coinhibitory signals and helps in better understanding the regulatory mechanisms of host immune suppression during leishmaniasis.

## Introduction

Leishmaniasis, caused by obligate intracellular protozoan parasites of the genus *Leishmania*, is endemic in more than 98 countries of tropical and temperate regions (1). The parasites are carried by 30 species of female sand fly that belongs to the genus *Phlebotomus* in the old world and *Lutzomyia* in the new world (2). About 20 species of *Leishmania* are responsible for three clinical forms, i.e., visceral leishmaniasis (VL), cutaneous leishmaniasis, and mucocutaneous leishmaniasis with an annual global prevalence of nearly 10 million cases and approximately 350 million people at risk (1). In view of limited drug options, widespread emergence of drug resistance, and prevalence of asymptomatic infections, it is widely recognized that vaccination could be an effective tool to control leishmaniasis (3, 4). However, there are no effective vaccines available against any form of human leishmaniasis. Killed *Leishmania* parasites and various native and recombinant vaccine antigens, such as gp63, gp46, m2, PSA2, TSA, LACK, LmsT1, Leish111f, etc., have been evaluated albeit none produced long-lasting protective immunity (5, 6). Acquisition of protective immunity following cure from clinical disease provided the basis for the feasibility of an effective vaccine but further elucidation of host immune mechanisms underlying protective immunity is essential.

*Leishmania* parasites adapt remarkable strategies to survive within the hostile environment of host. These parasites suppress effector properties of host phagocytic and non-phagocytic cells of innate immunity, which is characterized by decreased production of O_2_^−^, NO, H_2_O_2_, and pro-inflammatory cytokines such as IFNγ, IL-12, and TNFα (7). In addition, *Leishmania donovani* infections also inhibit proliferative abilities of CD4^+^ and CD8^+^ T cells and condition them to acquire a predominantly Th2 phenotype to produce anti-inflammatory cytokines such as TGFβ, IL-10 and IL-4 (8, 9). These results imply that an effective anti-leishmanial vaccine must promote Th1-biased immunity in the immunized host. Of the various vaccine formulations tested, live attenuated parasites allow host immune system to interact with a broad repertoire of antigens, which is considered to be essential for the development of protective immunity, and importantly cause no pathology. We have reported on centrin-deleted *L. donovani* parasites (LdCen^−/−^) as live attenuated vaccines in various animal models and in *ex vivo* human studies (10–14). LdCen^−/−^ parasites induce multiple cytokine (IFNγ, IL-2, and TNFα)-secreting Th1 cells in the immunized mice, a response shown to be the best predictor of protective immunity (11, 12, 15). In addition, protection induced by LdCen^−/−^ is long lasting, which further suggested strong induction of memory T cell responses by these parasites (11, 16). However, the initial interactions between the antigen-presenting cells (APCs) and the naïve T cells and the associated signals during T cell priming following immunization with LdCen^−/−^ parasites remain to be understood (17). Understanding the initial interactions that dictate the protection outcomes will reveal the immunological mechanisms important for an efficacious vaccine response.

Studies with viral antigens have suggested that the extent of opposing signals from costimulatory and coinhibitory molecules on the APCs determines the magnitude of T cell activation (18). The main function of immune inhibitory mechanisms is to maintain homeostasis between immune response (IR) and immune tolerance (IT) through cytokine-mediated extrinsic and intrinsic inhibitory processes (19). The extrinsic mechanisms involve recruitment of specialized effector cells such as T regulatory cells (Treg) that produce inhibitory (anti-inflammatory) cytokines such as IL-10 to counter activate the T cells (20). The intrinsic mechanisms are characterized by the expression of specialized receptors such as PD1, CTLA4, CD47, CD200, TIM3, and CD200R on the activated T cell surface, which deliver inhibitory signals *via* immunoreceptor tyrosine-based inhibitory motifs (ITIMs) and non-ITIMs after an interaction with the cognate ligand (18, 21). Studies on *Leishmania* reported an induction of PD1 and CTLA4, which after interaction with their ligands PDL1/2 and B7-2, respectively, inhibit T cell activation (20, 22, 23). However, the role of immune inhibitory signals particularly CD200–CD200R axis in *Leishmania* pathogenesis as well as vaccine-induced immunity remains unexplored.

CD200 is widely expressed on myeloid, lymphoid cell lineage, and non-immune cells and has a short cytoplasmic tail with no known signaling motifs (4, 24). Its receptor, CD200R, is shown to be differentially expressed on T cells, B cells, NK cells, and cells of myeloid origin (24–26). The cytoplasmic tail of CD200R recruits SH2-containing inositol phosphatase and RAS p21 protein activator 1 (RasGAP) that eventually dephosphorylate phosphatidylinositol 3 phosphate and RasGAP, which leads to deactivation of Ras-related kinases (27). The CD200–CD200R interactions have been shown to either negatively or positively regulate activated cells by means of pro- and anti-inflammatory cytokines, thereby maintaining a balance between IR and IT (21, 28, 29). Studies suggest that CD200–CD200R signaling negatively regulates IR, downregulates macrophage effector functions, inhibits antigen-specific T cell response, direct Th1 to Th2 transformation, and establishes IT in various tumors (30–32).

Studies with viral and bacterial pathogens showed that CD200–CD200R axis controls exacerbated inflammation during infection (26, 33, 34). In herpes (KSHV) infection, CD200 and its viral analog OX2 inhibit antigen-specific T cells, IFNγ production, and target-killing ability of the cytolytic granule component, CD107a (35). In HSV-1 infection, CD200 blockade suppressed Th1-type response and upregulated Treg cell production, suggesting the role of this axis in controlling T cell function and differentiation (36). A recent study by Cortez et al. (37) has shown that *L. amazonensis* induces CD200 expression and suppresses macrophage activation *via* iNOS inhibition that eventually leads to increased parasite growth. Despite the important role played by early immune regulatory signals in shaping the adaptive immunity, the role of immune inhibitory signals in vaccine-induced immunity has never been studied in leishmaniasis. This study is the first of its kind to understand the role of CD200–CD200R immune inhibitory axis in wild-type *L. donovani* pathogenesis and in live attenuated *L. donovani* vaccine-induced protective immunity.

## Materials and Methods

### Animals and Parasites

Six- to eight-week-old age-matched female C57Bl/6 mice and OT-II transgenic mice from the Charles River Laboratory were used in the experiments. The animal procedures and experiments described were approved by FDA’s Animal Care and Use Committee (Study 1995-26, updated and reapproved 8/18/2016). The animal program is fully compliant with the US PHS Policy on Humane Care and Use of Laboratory Animals and standards for full accreditation by AAALAC International. The wild-type (LdWT) and centrin1-deleted (LdCen^−/−^) lines of *L. donovani* were used (10). Virulence of the parasites is maintained by passaging through hamsters. The parasites were cultured according to the procedure previously described (11). In some experiments, LdWT expressing red fluorescent protein and LdCen^−/−^ expressing mCherry that both fluoresce in PE channel in flow cytometry were used (38). Centrin addback mutants (LdCen^−/−^AB) used in the study were cultured as described previously (10).

### Cultivation of Bone Marrow-Derived Dendritic Cells (BMDCs) and Parasites Infection

Bone marrow from the femurs and tibias of C57Bl/6 mice was collected and cultured with complete RPMI medium supplemented with 10% (v/v) FBS and gentamicin (20 µg/ml). Recombinant IL-4 and GM-CSF (20 ng/ml) were used to differentiate DCs for 7–8 days to obtain 80–90% purity of CD11c^+^ DCs that was verified by flow cytometry. BMDCs were harvested and plated on 24-well tissue culture plates. For early infection studies and measurements of CD200 expression and cytokines, DCs were infected with parasites at a ratio of 1:10 (DC:parasite) for 6 h at 37°C. Thereafter, DCs were thoroughly washed with PBS to remove non-internalized parasites. The number of amastigotes in infected DCs was stained with Diff-Quick (Baxter Healthcare Corporation) and parasite number was evaluated microscopically. The expression of CD200, number of intracellular amastigotes, and NO production were measured at 1, 4, 24, and 48 h post incubation of infected DCs following previously published protocols (11). Cytokines released by infected DCs were measured in culture supernatants collected at 24, 48, and 72 h post infection.

### Measurement of CD200 Expression

The CD200 expression levels in infected DCs *in vitro* were quantified by Western blotting using antiCD200 antibody. Briefly, DCs were lysed in lysis buffer containing protease inhibitors and CD200 recovered by coimmunoprecipitation using Pierce Cross-Link IP kit as per manufacturer instructions. The proteins were resolved on a 12% SDS-PAGE and detected on nitrocellulose membrane using α-CD200 primary and streptavidin-conjugated IR-linked secondary antibody (IRDye) on Odyssey imaging system (LI-COR, USA). The relative band density was quantified by Image Studio software (LI-COR, USA). *In vivo* expression of CD200 on the infected DCs was detected using LdWT^RFP^ or LdCen^−/−mCherry^ parasites. The mice were infected through tail vein with 3 × 10^6^ stationary phase LdWT^RFP^ or LdCen^−/−mCherry^ promastigotes. In each study, at least five mice were used per group. Age-matched naive mice were used as control. At 24 and 72 h post-infection, mice were sacrificed and infected DCs (CD11c^+^) from spleen and lymph nodes from different groups of mice were analyzed. Single-cell suspensions were prepared from spleens and lymph nodes, and RBCs were lysed using ACK lysing buffer. The cells were then labeled with AF700-α-CD3, BV785-α-CD4, BV650-α-CD8a, eFluor450-α-CD11c, and biotinylated α-CD200 PE–streptavidin. CD8a^−^CD11c^+^RFP/mCherry^+^ DCs from spleens and lymph nodes of mice infected with fluorescent parasites were selected on an LSR Fortessa flow cytometer, and the expression of CD200 on the infected DCs was estimated by biotinylated α-CD200 and streptavidin PE-Cy5.5 antibodies using FlowJo v10 software. In some experiments, we have isolated splenic DCs using miltenyi DC isolation kit. Mice spleens were collected and digested with collagenase (1 mg/ml) and DNase I (20 mg/ml) to make single-cell suspension. Splenocytes were labeled with APC-tagged α-TCRβ, α-NK1.1, α-CD19, and α-Ly6G Abs. α-APC magnetic beads were used and passed through the LS columns to select specific cell types. Flow through enriched DC population was collected and plated before infection with LdWT, LdCen^−/−^, or LdCen^−/−^AB parasites. CD200 expression was also measured at 1 and 2 weeks post infection following similar procedures above by flow cytometric analysis.

### Multiplex Cytokines ELISA and NO Quantification

Cultured supernatant obtained from DCs at respective time points were used to measure cytokines by MILLIPLEX mouse cytokine/chemokine magnetic bead panel (Millipore). The staining plate was prepared according to the manual and read in a Luminex-100 (Luminex) system using Bio-Plex manager software 5.0. The levels of cytokines were determined by a standard curve for each specific cytokine. NO (nitrite/nitrate) production was determined by the Griess reaction kit (Sigma-Aldrich) in culture supernatant (11).

### BMDCs and T Cell Coculture Studies: Measurement of OTII Cells Proliferation and Cytokines Production

For T cell coculture assays, DCs were harvested and pulsed with 2 µg/ml OVA peptide (323–339, AnaSpec) for 4 h. After OVA pulsing, DCs were infected with LdWT and LdCen^−/−^ promastigotes for 6 h, and then thoroughly washed to remove non-internalized parasites and further incubated for 18 h. Parasite-infected OVA-pulsed DCs were cocultured with CD4^+^ T cells isolated from OT-II transgenic mice (Jackson Laboratory). CD4^+^ T cells from OT-II transgenic C57Bl/6 mice were incubated in 5 µM CFSE (Molecular Probes/Invitrogen) for 10 min in RPMI 1640 without FCS, followed by 5 min of quenching in ice-cold RPMI 1640 plus 10% FCS and subsequently washed thoroughly before plating in 96-well tissue culture plates at the ratio of 1:10 (BMDCs to T cells) per well. Cells were cultured for 5 days at 37°C with 5% CO_2_, harvested, washed, and stained for cytokines as described in Section “Flow Cytometry: Surface and Intracellular Staining.” The rate of proliferation and cytokines production by OT-II cells in coculture experiment were measured in the presence and absence of α-CD200 antibody (10 µg/ml) by flow cytometry. CFSE staining of OT-II T cells was performed as described previously (38). Appropriate isotype controls were used as needed (Purified IgG2a, κ Isotype Control, BD Clone R35-95). Following coculture, the cells were blocked with rat α-mouse CD16/32 (1 µg/10^6^ cells) for 20 min at 4°C. Cells were then surface stained with AF-700-α-CD3, BV-785-α-CD4, BV-650-α-CD8a, FITC-α-CD44, PE-α-CD200, and APC-α-CD200R antibodies for 30 min (each with 1:200 dilution; 4°C or ice). The cells were then stained with LIVE/DEAD Fixable Aqua (Invitrogen/Molecular Probes) to mark dead cells. For intracellular cytokines staining, OT-II cells were washed with wash buffer and fixed with the cytofix/cytoperm kit (BD Biosciences) for 20 min (room temperature). Intracellular staining was done with PE-Cy7-α-IFNγ, eFluor450-α-TNF, APC-α-IL-2, and PerCPCy5.5-α-IL-10 for 30 min (each with 1:300 dilution; 4°C or ice). Stained cells were acquired on LSR Fortessa flow cytometer, and the data were analyzed using FlowJV10.

### CD200R Expression on CD4^+^ T Cells

C57Bl/6 mice were immunized *via* tail vein with 3 × 10^6^ stationary phase LdCen^−/−^ promastigote parasites. Similarly, mice were infected with wild-type *L. donovani* (LdWT) stationary phase promastigotes. Another group of age-matched mice (naïve controls) received a saline solution (PBS). Animals were sacrificed on days 7 and 14 post immunization to assess the CD200R expression on CD4^+^ T cells and their functional characteristics by flow cytometry following procedures described above (38). In each group, 6–8 animals were used in each experiment. In independent experiments, mice infected with LdWT parasites were treated with α-CD200 or isotype control antibodies. Expression of CD200R on the activated CD4^+^ T cells was measured at 21 days post infection.

### Blocking CD200 in Mice Using α-CD200 Antibodies

To validate if CD200 blocking in LdCen^−/−^-immunized animals provides better protection in terms of reduced spleen parasite load and increased CD4^+^ multifunctional T cell response in *Leishmania* infection, we treated LdCen^−/−^ immunized mice with α-CD200 antibody. Briefly, the mice were immunized *via* tail vein with 3 × 10^6^ stationary phase centrin gene knockout (LdCen^−/−^) promastigote parasites. An independent group of LdCen^−/−^ immunized mice were treated with α-CD200 antibodies (20 µg/kg of body weight), on days 0, 3, 6, 9, and 12. Another group of age-matched mice (naïve controls) received a saline solution (PBS). After 12 weeks, animals were infected with 10^5^ virulent metacyclic promastigotes, which were isolated from stationary phase culture of wild-type Ld1S (LdWT) strain by Ficoll density gradient separation as described elsewhere (38). After 9 weeks of challenge, animals were sacrificed to measure spleen parasite load and multifunctional T cells responses induced by the LdCen^−/−^ immunization. To measure the impact of blocking CD200 expression on the wild-type LdWT infection, in separate experiments two groups of mice were infected with virulent metacyclic promastigotes (10^5^) of which one group was challenged with α-CD200 antibodies as described earlier. These two groups of animals were sacrificed after 4 weeks and spleen parasite burden was estimated.

### Flow Cytometry: Surface and Intracellular Staining

Spleens of immunized and control animals were removed on scheduled time points, and splenocytes were obtained by maceration and passing through 70 µm filter following ACK lysis as described above. Cells were used for surface and intracellular cytokines staining either in the absence or presence of leishmanial soluble antigens (SLA, 80 µg/ml) (11). Following overnight culture in the presence of SLA in complete RPMI 1640 medium at 37°C, the cells were blocked with protein transport inhibitor (BD GolgiStop; BD Pharmingen) for 4 h at 37°C. Before surface staining, the cells were blocked with rat α-mouse CD16/32 (1 µg/10^6^ cells) for 20 min at 4^0^C. Cells were surface stained with AF700-α-CD3, BV785-α-CD4, BV650-α-CD8a, FITC-α-CD44, and PerCPCy5.5-αCCR7 antibodies for 30 min (each with 1:200 dilution; 4°C or ice). The cells were then stained with LIVE/DEAD Fixable Aqua (Invitrogen/Molecular Probes) to mark dead cells. For intracellular cytokines staining, cells were washed with wash buffer and fixed with the cytofix/cytoperm kit (BD Biosciences) for 20 min (room temperature). Intracellular staining was done with PE-Cy7-αIFNγ, eFluor450-α-TNF, APC-α-IL-2, and PerCPCy5.5-α-IL-10 for 30 min (each with 1:300 dilution; 4°C or ice). All antibodies were from BD Biosciences, except for CD200 and CD200R (eBiosciences). Cells were acquired either on an LSR Fortessa or LSR X20 (BD Biosciences) equipped with required laser lines using DIVA 6.1.2 software. Data were analyzed with FlowJo software version 10 (TreeStar). For analysis, first doublets were removed using width parameter; dead cells were excluded based on staining with the LIVE/DEAD Aqua dye. Lymphocytes were identified according to their light-scattering properties. CD4^+^ T cells were identified as CD3^+^ lymphocytes uniquely expressing either CD4^+^ or CD8^+^. Upon further gating, CD200 and CD200R were measured in CD4^+^CD44^hi^ T cells. Fluorescence minus one controls were used for proper gating of positive events for designated cytokines.

### Statistical Analysis

Statistical analysis of differences between mean values of groups was determined by parametric unpaired two-tailed Student *t*-test and non-parametric Mann–Whitney test on GraphPad Prism 5.0 software. A *p* value < 0.05 was considered significant.

## Results

### LdCen^−/−^ Infection Induces Comparatively Low Expression of CD200 on BMDCs

**Figure 1:**
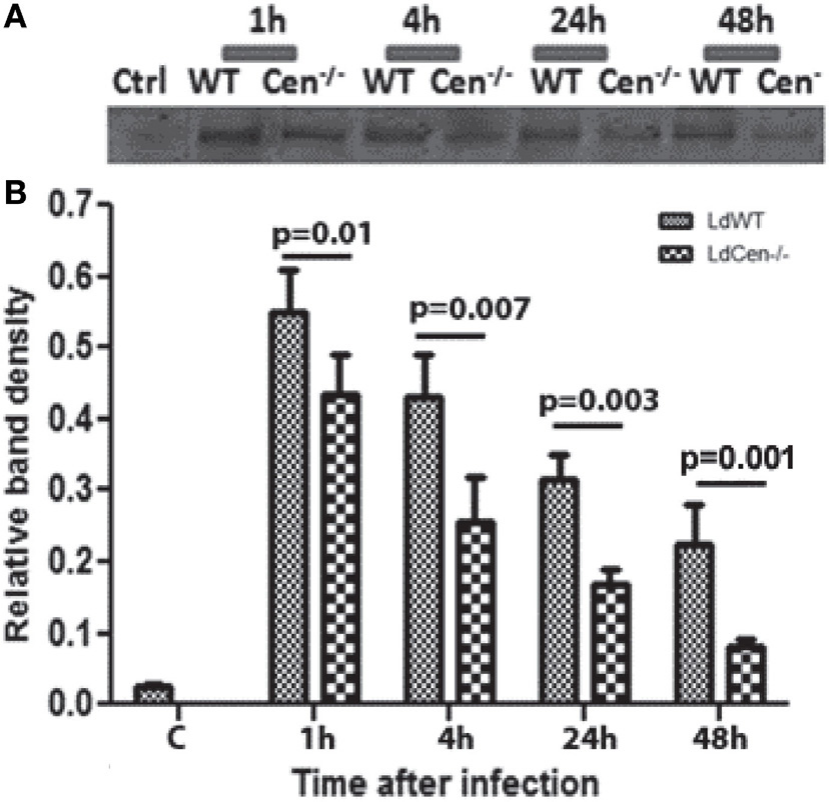
Expression of CD200 (upper panel) and relative band density (lower panel) in wild-type (LdWT) and centrin gene-deleted (LdCen^−/−^) parasite-infected DCs at different time points post infection **(A)**. Bone marrow-derived dendritic cells were differentiated in the presence of GM-CSF and IL-4, cultured in 24-well tissue culture plate, and infected with LdWT and LdCen^−/−^ parasites at a ratio of 1:10 (DC:parasite). CD200 expression was measured by coimmunoprecipitation with an α-CD200 antibody followed by immunoblotting at 1, 4, 24, and 48 h **(B)**. The CD200 expression was induced by both parasites, but the level of expression was comparatively low in LdCen^−/−^ parasites at each time point.

To analyze the expression of CD200 on DCs upon infection with LdWT or LdCen^−/−^ parasites, BMDCs (>85% CD11c^+^) were infected with these parasites *in vitro*. The CD200 expression was measured after 1, 4, 24, and 48 h post infection in DC lysates. The expression of CD200 was found to be significantly low (*p* ≤ 0.05) in LdCen^−/−^-infected DCs at all time points compared to LdWT-infected DCs (Figures 1A,B, immunoblot and densitometry plot). Furthermore, the level of CD200 expression in both infections was at a maximum at early infection stages, which declined at later time points. The expression of CD200 was consistently downregulated in LdCen^−/−^-infected DCs, and at 48 h the expression was significantly (*p* = 0.001) less than that in LdWT-infected DCs. We did not observe any significant difference in the percentage of infected DCs by both parasites at all time points which confirmed the equivalent infectivity of LdWT and LdCen^−/−^ parasites (Figure S1A in Supplementary Material). As expected, a gradual increase in the number of parasites was observed in LdWT-infected DCs at both 24 h (*p* = 0.0113) and 48 h (*p* = 0.0155) post infection (Figure S1B in Supplementary Material). The parasite number in LdCen^−/−^-infected DCs was increased up to 24 h post infection but remained same till 48 h post infection (Figure S1B in Supplementary Material). Furthermore, the level of NO production was found comparatively more in LdCen^−/−^-infected DCs that was significant at 4 h (*p* = 0.0048) and 24 h (*p* = 0.0376) post infection (Figure S1C in Supplementary Material). At 48 h time point, the NO level was still high in LdCen^−/−^ compared to LdWT infection, but the difference was not statistically significant (Figure S1C in Supplementary Material). We also measured the levels of pro-inflammatory cytokines IFNγ, TNFα, IL-12, and IL-1β in cultured supernatant collected after 24, 48, and 72 h post infection to verify if the levels of these cytokines have any correlation with CD200 expression in parasite-infected DCs. The levels of IL-12 (*p* = 0.0101) and TNFα (*p* = 0.0365) were found to be elevated at 48 h post infection in LdCen^−/−^-infected DCs (Figures S2A,B in Supplementary Material). However, at each time points there was no difference in the IFNγ level between WT and LdCen^−/−^-infected DCs (Figure S2C in Supplementary Material), whereas the IL-1β level was significantly more (*p* = 0.008) in LdCen^−/−^-infected DCs post 72 h infection (Figure S2D in Supplementary Material).

### LdCen^−/−^ Parasite Infection Limits CD200 Expression on Infected Cells Including CD11c^+^ DCs *In Vivo* Compared to LdWT Parasites

**Figure 2:**
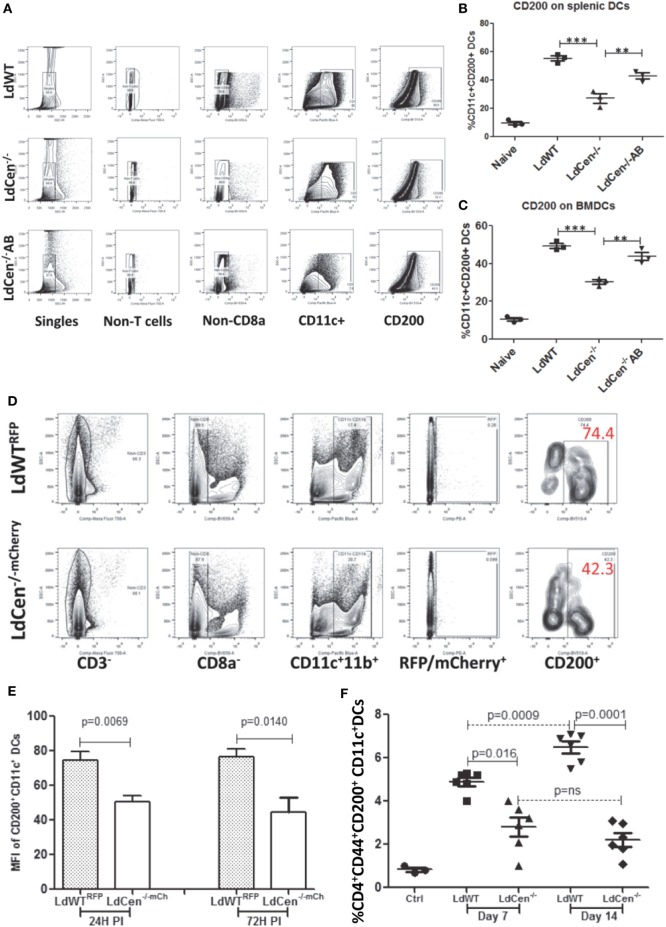
Percentage of CD200^+^ dendritic cells (CD11c^+^ DCs) *in vitro* and in the infected animals after 24 and 72 h post infection. **(A)** A schematic representation of flow cytometry analysis followed to measure CD200^+^ dendritic cells. **(B)** Expression of CD200 on the splenic DCs infected *in vitro* with LdWT, LdCen^−/−^, or LdCen^−/−^AB parasites. **(C)** Expression of CD200 on the bone marrow-derived dendritic cells (BMDCs) differentiated and infected *in vitro* with LdWT, LdCen^−/−^, or LdCen^−/−^AB parasites. A representative data set from duplicate biological repeat experiments is shown. **(D)** The gating strategy to assess the expression of CD200 on parasitized DCs and from LdWT^RFP^ or LdCen^−/−mCherry^-infected mice is shown. Each group contained five mice, and this *in vivo* experiment was repeated at least four times with similar results. **(E)** At early infection time points (24 and 72 h), the LdWT parasites induced significantly high CD200 expression on CD11c^+^ DCs compared to LdCen^−/−^ infection. **(F)** Percentage of CD200^+^ dendritic cells (CD11c^+^ DCs) in the spleen of infected animals on days 7 and 14. At the indicated time points, animals were sacrificed and CD200 expression was measured on CD11c^+^ DCs obtained from spleen cells.

To test whether downregulation of CD200 expression also occurs in splenic DCs due to LdCen^−/−^ infection, we infected splenic DCs isolated from C57Bl/6 mice with either LdWT or LdCen^−/−^ *in vitro*. Flow cytometric analysis showed that 24 h post infection, CD11c^+^ splenic DCs showed a strong induction of CD200 in LdWT and a reduced CD200 expression in LdCen^−/−^ (Figures 2A,B). Furthermore, infection with an addback mutant of LdCen^−/−^ re-expressing centrin induced a higher level of CD200 compared to LdCen^−/−^ infection (Figure 2B). Similarly, infection of BMDCs with LdCen^−/−^ add back mutant induced CD200 expression to levels observed in LdWT infection (Figure 2C), whereas LdCen^−/−^-infected BMDCs showed reduced expression of CD200 compared to LdWT. To further assess the expression of CD200 at earlier time points *in vivo* and to select bonafide-infected cells, we performed mouse infection experiments with fluorescent *Leishmania* parasites. Use of LdWT^RFP^ or LdCen^−/−mCherry^ parasites enabled us to isolate parasitized cells (Figure 2D) as early as 24 h from the spleens and lymph nodes of infected mice. The parasitized cells were identified as CD3^−^CD8a^−^CD11c^+^CD11b^+^RFP/mCherry^+^ cells that include DCs. Consistent with the *in vitro* data, immunization with LdCen^−/−^ parasites induced significantly less CD200 expression on the DCs compared to LdWT infection at both 24 h (*p* = 0.0069) and 72 h (*p* = 0.014) (Figure 2E). To analyze if reduction of CD200 expression is transient or sustained over time in LdCen^−/−^ infection *in vivo*, we examined splenic CD11c^+^ DCs at days 7 and 14 in LdWT and LdCen^−/−^-infected animals. On both days, significantly less CD200 expressing DCs were found at day 7 (*p* = 0.016) and at day 14 (*p* = 0.0001) in LdCen^−/−^-infected animals compared to LdWT-infected animals (Figure 2F). In addition, the percentage of CD200 expressing DCs was more (*p* = 0.0009) on day 14 compared to day 7 in LdWT-infected animals, whereas there was no significant change in CD200^+^ DCs in LdCen^−/−^-immunized animals. The suppression of CD200 correlated with the parasite burden which was significantly less on both days (day 7, *p* = 0.0087; day 14, *p* = 0.0067) in LdCen^−/−^-immunized mice (Figure S3 in Supplementary Material), further suggesting that immunization by LdCen^−/−^ parasites is followed by early control of parasite burden.

### *In Vitro* Blocking of CD200 on DCs Infected With LdWT and LdCen^−/−^ Parasites Resulted in Enhanced Proliferation and Cytokines Production Abilities of OT-II CD4^+^ T Cells

**Figure 3:**
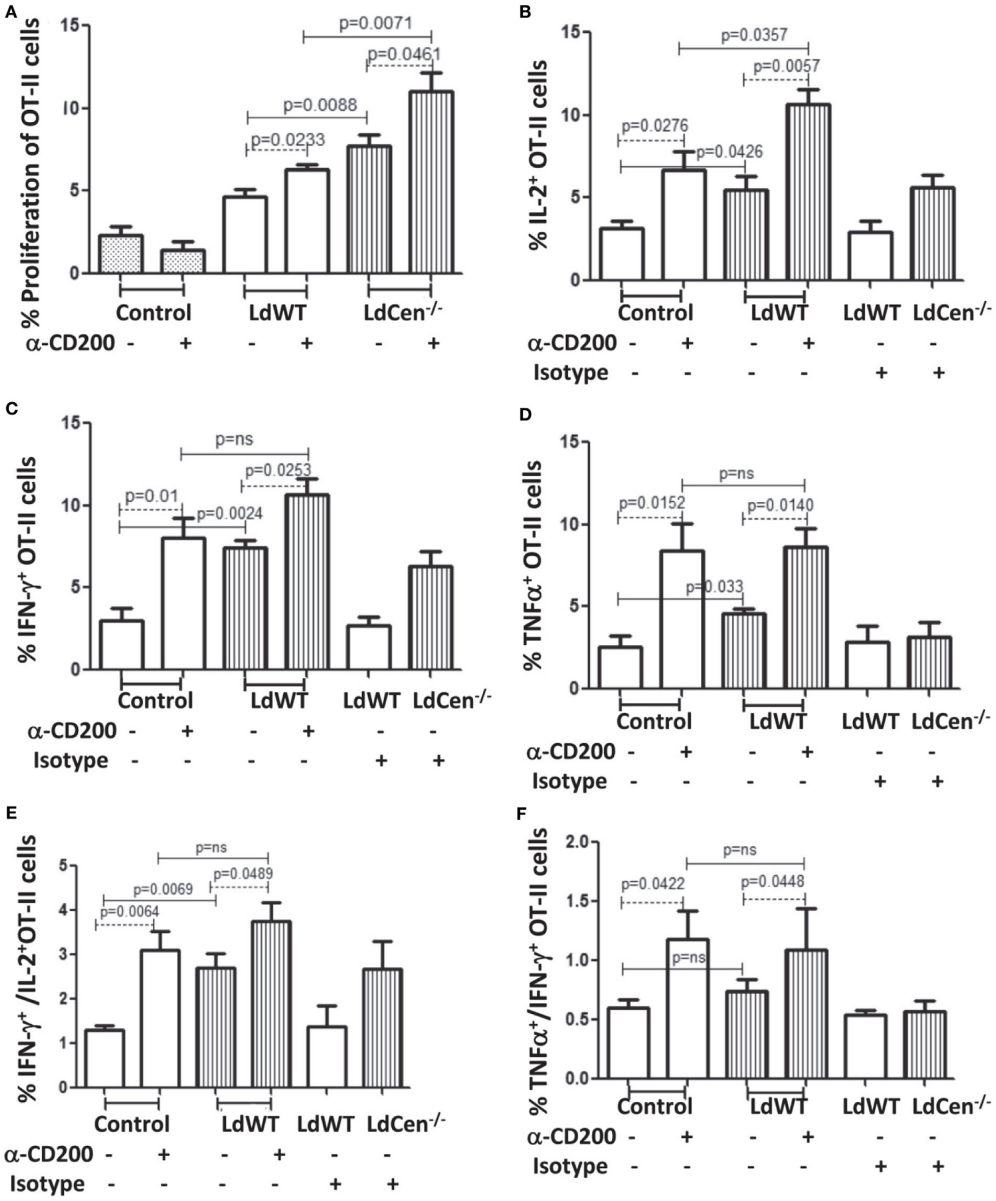
Rate of proliferation and cytokine production by OT-II T cells, cocultured with LdWT and LdCen^−/−^-infected DCs before and after α-CD200 blocking. DCs were pulsed with 2 µg/ml OVA peptide for 4 h, infected by LdWT and LdCen^−/−^ parasites for 6 h. Parasite-infected OVA-pulsed DCs were cocultured with CFSE (5 µM)-stained OT-II cells for 5 days at 37°C with 5% CO_2_. Rate of proliferation and cytokines release by OT-II cells were measured by flow cytometry. **(A)** α-CD200 blocking significantly (LdWT, *p* = 0.0233; LdCen^−/−^, *p* = 0.0461) induced OT-II cell proliferation in both groups. The rate of proliferation with and without α-CD200 antibodies is shown. **(B)** The CD200 blocking significantly (*p* < 0.05 in all conditions) increased the percentage of OT-II cells producing **(B)** IL-2, **(C)** IFN-γ, and **(D)** TNFα cytokines in both LdWT and LdCen^−/−^ parasites as compared to those that were cultured without α-CD200 antibody. **(E)** The multiple cytokines (IFN-γ and IL-2), and **(F)** TNFα and IFN-γ-producing abilities of OT-II cells were also induced after CD200 blocking in both LdWT and LdCen^−/−^ groups. A representative data set from duplicate biological repeat experiments is shown.

CD200 is a ligand for CD200R that controls T cell proliferation and functions through inhibitory signals mediated upon CD200–CD200R ligation (26). Therefore, we first analyzed the effect of CD200 expression on DCs upon parasite infection on proliferation and function of T cells. We also analyzed the effect of blocking CD200 expression by using α-CD200 antibodies. Ova peptide-treated parasite-infected DCs were cocultured with CFSE-stained OT-II cells either in the presence or absence of α-CD200 antibodies. Blocking of CD200 expression on DCs significantly induced the proliferation of CD4^+^ T cells in both LdWT (*p* = 0.0233) and LdCen^−/−^ (*p* = 0.0461) groups (Figure 3A). The proliferation of OT-II cells was comparatively high in the LdCen^−/−^ group either with (*p* = 0.0071) or without (*p* = 0.0088) α-CD200 antibodies compared to the LdWT group. We further measured intracellular levels of IL-2, a marker of T cell proliferation, and IFNγ and TNFα-producing abilities of OT-II cells (Figures 3B–D). Blocking of CD200 significantly induced the proportion of OT-II cells producing IL-2 (Figure 3B; LdWT ± α-CD200, *p* = 0.0276; LdCen^−/−^ ± α-CD200, *p* = 0.0057), IFNγ (Figure 3C, LdWT ± α-CD200, *p* = 0.01; LdCen^−/−^ ± α-CD200, *p* = 0.0253), and TNFα (Figure 3D, LdWT ± α-CD200, *p* = 0.0152; LdCen^−/−^ ± α-CD200, *p* = 0.014) compared to those that were cultured without α-CD200 antibody. Between the LdWT and LdCen^−/−^ groups, the percentage of IL-2 (Figure 3B, *p* = 0.0426), IFNγ (Figure 3C, *p* = 0.0024), and TNFα (Figure 3D, *p* = 0.033) producing OT-II cells was high in the LdCen^−/−^ infected group. Furthermore, the multiple cytokine-producing abilities of OT-II cells were also induced following CD200 blocking in the LdWT and LdCen^−/−^ groups (Figures 3E,F). OT-II cells expressing both IFNγ and IL-2 significantly increased in LdWT (Figure 3E, *p* = 0.0064) and LdCen^−/−^ (Figure 3E, *p* = 0.0489) infections upon blocking with α-CD200 antibodies. Similarly, OT-II cells expressing both TNFα and IFNγ significantly increased in LdWT (Figure 3F, *p* = 0.0422) and LdCen^−/−^ (Figure 3F, *p* = 0.0448) infections upon blocking with α-CD200 antibodies. Isotype antibodies were used as controls to demonstrate the specific effects of CD200 antibodies (Figures 3B–F).

### LdCen^−/−^ Infection Suppresses Expression of CD200R on Antigen-Experienced CD4^+^ T Cells (CD4^+^CD44^+^) as Compared to LdWT Parasite Infection *In Vivo*

**Figure 4:**
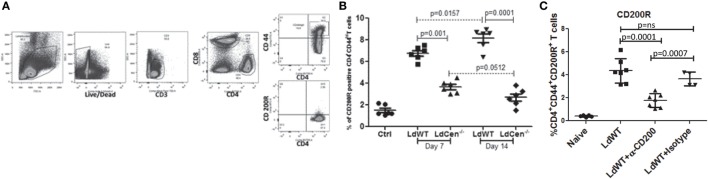
Expression of CD200R on antigen-experienced (CD44^hi^) CD4 T cells in LdWT and LdCen^−/−^ immunized animals. **(A)** The gating strategy for analyzing CD200R expression is shown in an illustrative flow cytometric analysis. The CD200R^+^ populations were analyzed on CD4^+^ T cells expressing CD44^hi^ belonging to CD3^+^ T cells population. **(B)** The expression of CD200R in LdWT or LdCen^−/−^-infected animals on days 7 and 14 is shown. The data set represents duplicate biological repeat experiments with 5–6 animals per group. **(C)** Expression of CD200R on the activated CD4^+^ T cells in LdWT-infected mice untreated or treated with either α-CD200 or isotype control antibodies 21 days post infection is shown. The data set represents duplicate biological repeat experiments with similar results.

We hypothesized that if expression of CD200 is low in LdCen^−/−^ immunized animals relative to LdWT infection, the expression level of its receptor CD200R correspondingly would also be restrained. To test this hypothesis, the percentage of CD200R^+^ populations was measured as CD3^+^CD4^+^CD44^+^CD200R^+^ T cells on days 7 and 14 post infection (Figures 4A,B). Results showed significantly less CD4^+^CD44^+^CD200R^+^ T cells in LdCen^−/−^-immunized animals on both time points (day 7, Figure 4B, *p* = 0.001; and at day 14, *p* = 0.0001) compared to LdWT infection. In LdCen^−/−^-immunized animals, percentage of CD200R expressing T cells did not change between days 7 and 14 (*p* = 0.0512). In contrast, in LdWT-infected animals the percentage of CD200R^+^ CD4^+^ T cells increased with time (*p* = 0.0157). To measure the impact of blocking CD200 on CD200R expression, we injected CD200 blocking antibodies or an isotype control on days 0, 3, 6, 9, and 12 following infection with LdWT parasites. Flow cytometric analysis 21 days post infection showed that expression of CD200R on activated CD4^+^ T cells was reduced upon treatment with α-CD200 antibodies but not in isotype-treated controls indicating the specificity of the CD200 blocking antibodies (Figure 4C).

### Expression of CD200R by CD4^+^CD44^+^ T Cells Is Correlated With Induction of IL-10 Production

**Figure 5:**
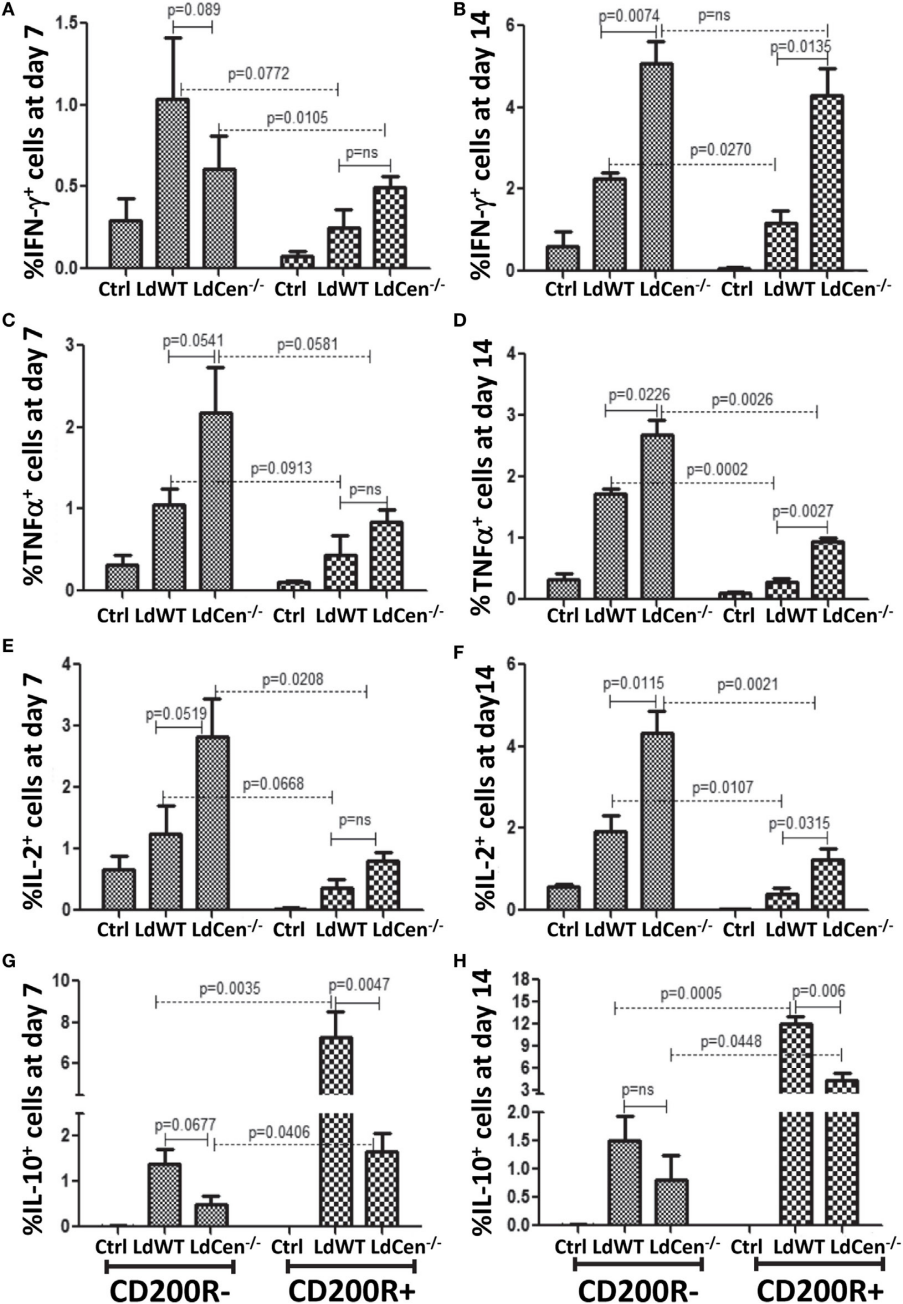
Expression of IFNγ, TNFα, IL-2, and IL-10 in CD4^+^CD44^+^CD200R^−^ and CD^+^CD44^+^CD200R^+^ T cells in LdWT- and LdCen^−/−^-infected mice at days 7 and 14. The percentage of CD200R^−^ cells producing **(A)** IFNγ, **(C)** TNFα, and **(E)** IL-2 on day 7 post infection in LdWT- and LdCen^−/−^-infected mice. The percentage of CD200R^−^ cells producing **(B)** IFNγ, **(D)** TNFα, **(F)** and IL-2 on day 14 post infection in LdWT- and LdCen^−/−^-infected mice. Percentage of cells expressing IL10 in both CD200R^−^ and CD200R^+^ T cells at both day 7 **(G)** and day 14 **(H)**, following infections with either LdWT or LdCen^−/−^ parasites. Data are representative of biological repeat experiments with at least four animals in each group.

Since our *in vitro* and *in vivo* experiments showed that the levels of CD200 on parasitized DCs and CD200R on the activated CD4^+^ T cells are distinctly different in LdWT and LdCen^−/−^ infections, we wanted to analyze whether the level of CD200R expression on T cells leads to differential functional phenotype. Toward this, we isolated CD200R^−^ and CD200R^+^ T cells and measured the frequency of the CD4^+^ T cells producing IFNγ, TNFα, and IL-10 individually or a combination of such cytokines (Figure 5). On day 7 post infection, a comparison of CD200R^−^ and CD200R^+^ CD4^+^ T cells revealed that the percentage of CD200R^−^ cells producing IFNγ (Figure 5A, LdWT, *p* = 0.0772; LdCen^−/−^, *p* = 0.0105), TNFα (Figure 5C, LdWT, *p* = 0.0913; LdCen^−/−^, *p* = 0.0581), and IL-2 (Figure 5E, LdWT, *p* = 0.0668; LdCen^−/−^, *p* = 0.0208) were higher than those T cells that were CD200R^+^ but not always statistically significant. At day 14 post infection, only TNFα (Figure 5D LdWT, *p* = 0.0002; LdCen^−/−^, *p* = 0.0026) and IL-2 (Figure 5F, LdWT, *p* = 0.0107; LdCen^−/−^, *p* = 0.0021) producing CD200R^−^ T cells were significantly higher than CD200R^+^ T cells. However, there was no significant difference in IFNγ-producing cells in LdCen^−/−^ infection (Figure 5B) even though significant differences were found in the LdWT group (Figure 5B, *p* = 0.0270). Between LdWT and LdCen^−/−^ groups, the IFNγ, TNFα, and IL-2 secreting CD200R^−^ T cells were found significantly more in LdCen^−/−^-immunized animals at both time points (Figures 5A–F, with *p* < 0.05 for all three pair-wise comparisons) except for IFNγ on day 7 (Figure 5A). In LdCen^−/−^ immunized animals, the CD200R^+^ cells were also found to produce more IFNγ (Figure 5B, *p* = 0.0135), TNFα (Figure 5D, *p* = 0.0027), and IL-2 (Figure 5F, *p* = 0.0315) at day 14 as compared to LdWT.

Importantly, a comparison between CD200R^−^ and CD200R^+^ T cells showed that the percentage of IL-10-producing CD200R^+^ T cells was significantly more on both day 7 (Figure 5G, LdWT, *p* = 0.0035; LdCen^−/−^, *p* = 0.0406) and day 14 (Figure 5H, LdWT, *p* = 0.0005; LdCen^−/−^, *p* = 0.0448) in LdWT-infected animals compared to LdCen^−/−^-immunized animals. IL-10^+^ CD200R^−^ T cells were also found more in LdWT-infected animals compared to LdCen^−/−^-immunized animals on both days 7 and 14 (Figures 5G,H).

### Expression of CD200R on Antigen-Experienced CD4^+^ T Cells Resulted in the Loss of Ability to Produce Multiple Cytokines

**Figure 6:**
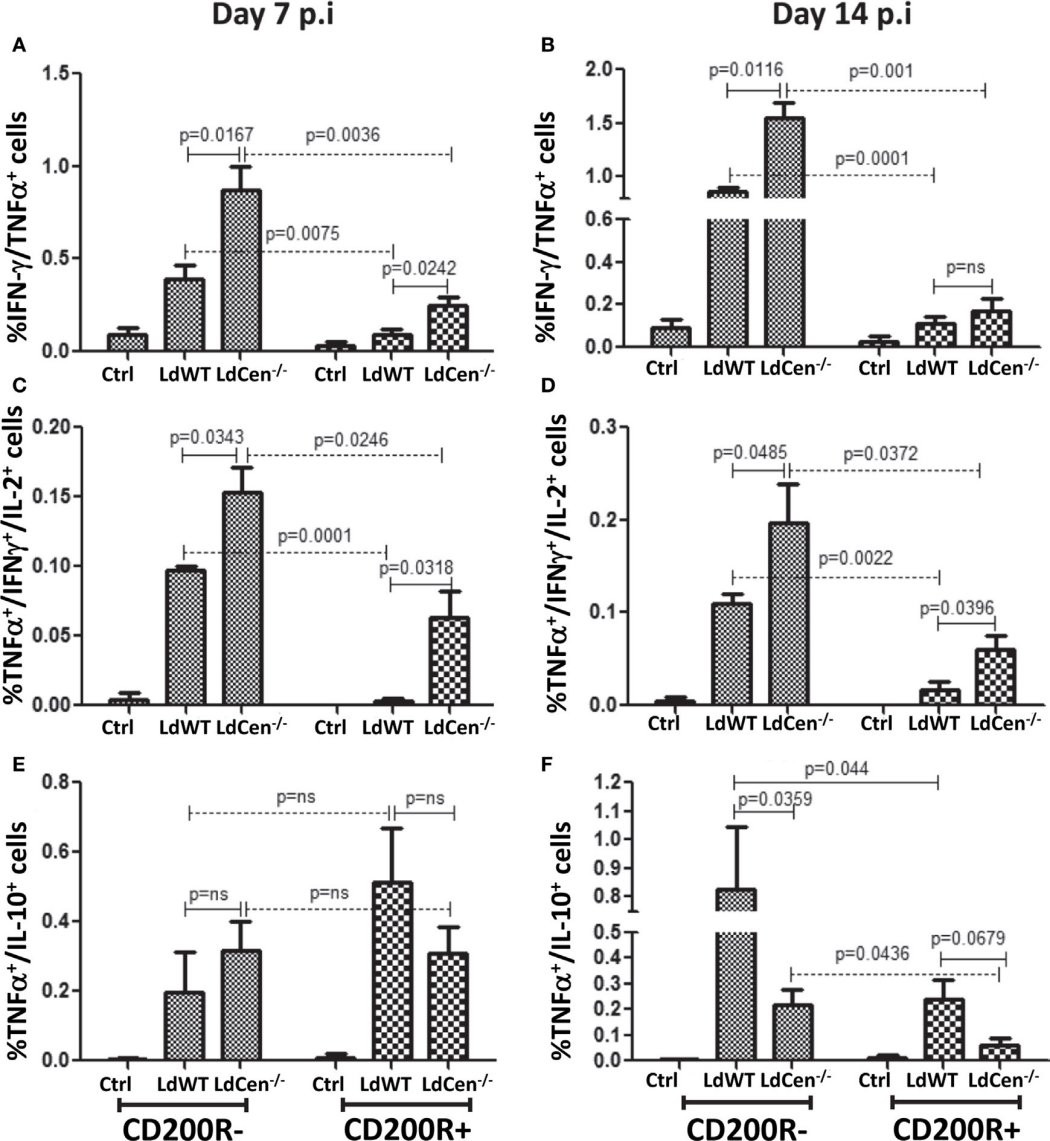
Percentage of multiple cytokines secreting CD4^+^CD44^+^CD200R^−^ and CD^+^CD44^+^CD200R^+^ T cells in LdWT- and LdCen^−/−^-challenged mice at days 7 and 14. The frequency of CD4^+^CD44^+^CD200R^+^ and CD4^+^CD44^+^CD200R^−^ T cells producing multiple Th1 cytokines [IFNγ/TNFα; double positives (DP), IFNγ/TNFα/IL-2, triple positive (TP)] was measured at both time points. **(A)** IFN-γ/TNF-α DP cells in CD4^+^CD44^+^CD200R^−^ and CD4^+^CD44^+^CD200R^+^ T cell populations on day 7 and **(B)** on day 14. **(C)** TNF-α/IFN-γ//IL-2 TP cells in CD4^+^CD44^+^CD200R^−^ and CD4^+^CD44^+^CD200R^+^ T cell populations on day 7 and **(D)** on day 14. **(E)** TNF-α/IL-10 DP cells in CD4^+^CD44^+^CD200R^−^ and CD4^+^CD44^+^CD200R^+^ T cell populations on day 7 and **(F)** on day 14. Data are representative of biological repeat experiments with at least four animals in each group.

Previous studies suggested that increased expression of CD200R on activated CD4^+^ T cells due to chronic infection results in the loss of their multifunctional potential (38). Since a multi-functional T cell response is the best known predictor for protection against *Leishmaniasis*, and was demonstrated earlier by us using LdCen^−/−^ immunization (11, 12), we were interested in investigating whether early CD200R expression determines the potential for multi-functionality in a vaccine-induced IR. Accordingly, the percentages of IFNγ/TNFα double positive (DP) and IFNγ/TNFα/IL-2 triple positive (TP) Th1 cytokine-producing CD4^+^CD44^+^CD200R^−^ T cells were significantly higher at day 7 (Figure 6A DP: LdWT *p* = 0.0075, LdCen^−/−^ *p* = 0.0036; Figure 6C, TP: LdWT *p* = 0.0001, LdCen^−/−^ *p* = 0.0246) compared to CD4^+^CD44^+^CD200R^+^ T cells. Similar differences between CD200R^+^ and CD200R^−^ T cells were also observed on day 14 (Figure 6B, DP: LdWT *p* = 0.0001, LdCen^−/−^ *p* = 0.001; Figure 6D; TP: LdWT *p* = 0.0022, LdCen^−/−^ *p* = 0.0372). However, in LdCen^−/−^-immunized animals, the percentage of CD200R^−^ DP/TP T cells was found significantly more (Figures 6A–D, *p* < 0.05 for all pair-wise comparisons) than that of LdWT-infected animals. The CD200R^+^ DP/TP T cells were also found to be comparatively more in LdCen^−/−^-immunized animals at both days 7 and 14 post infection. On the other hand, the percentage of IL-10/TNFα DP CD200R^−^ and CD200R^+^ T cells was found more in LdWT-infected animals as compared to LdCen^−/−^-immunized animals only at day 14 and not at day 7 (Figures 6E,F).

### *In Vivo* Blocking of CD200 by α-CD200 Antibody Resulted in Increased Percentage of CD4^+^CD44^+^ T Cells Producing Th1 Cytokines

**Figure 7:**
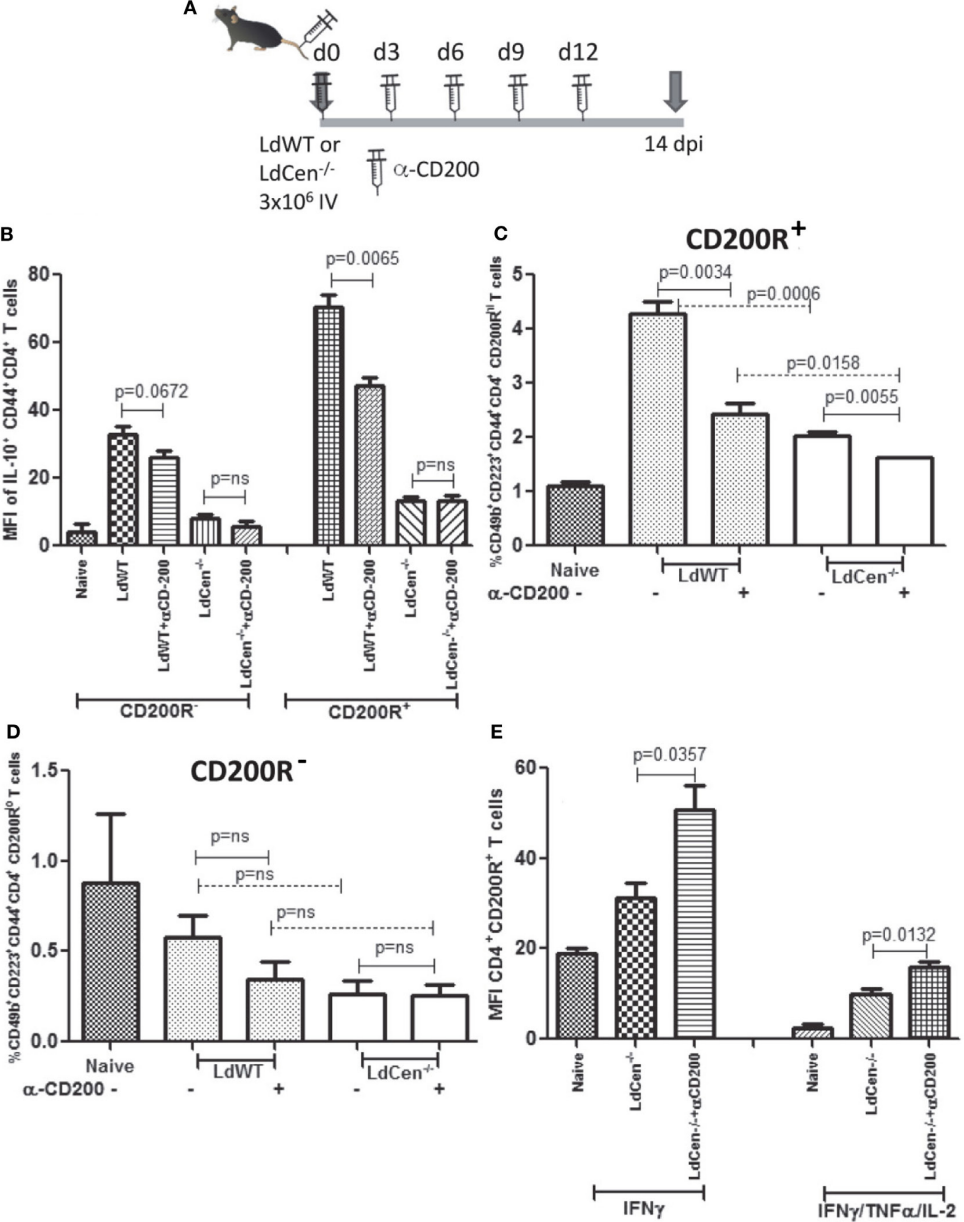
Effect of CD200 blocking on the function of CD^+^CD44^+^CD200R^+^ T cells in LdCen^−/−^ immunized and virulent LdWT parasite-infected mice. **(A)** Schematic showing the schedule of infections and administration of α-CD200 antibodies. Animals were immunized and treated with α-CD200 antibodies on the days indicated. **(B)** IL-10 producing CD200R^−^ and CD200R^+^ T cell populations are shown. **(C)** CD200R^+^ (CD200R hi panel) cells expressing CD49B and CD223 markers representing Tr1 populations in LdWT and LdCen^−/−^-infected and/or α-CD200-treated mice are shown. **(D)** CD200R^−^ (CD200R low panel) cells expressing CD49B and CD223 markers representing Tr1 populations in LdWT and LdCen^−/−^-infected and/or α-CD200-treated mice are shown. **(E)** CD4^+^CD44^+^CD200R^+^ T cells expressing IFN-γ alone or multifunctional cytokine response IFNγ/TNFα/IL-2 after CD200 blocking in LdCen^−/−^ parasite-infected animals are shown. Data are representative of biological repeat experiments with at least four animals in each group.

To test whether CD200–CD200R signaling indeed determines the multifunctional response, we evaluated if blocking of CD200-mediated signaling *in vivo* could alter the Th2-type response induced by LdWT infection and conversely enhance the vaccine-induced response in LdCen^−/−^-immunized animals during T cell priming. Subdued induction of CD200 by LdCen^−/−^ compared to LdWT infection suggested that blocking this signaling could divert more CD4^+^ T cells to acquire multi-functionality. We infected mice with either LdWT or LdCen^−/−^ and treated with α-CD200 antibodies on days 0, 3, 6, 9, and 12 and analyzed the early IR (Figure 7A). Results showed that consistent with our *in vitro* blocking results, *in vivo* blocking with α-CD200 antibodies resulted in a significant reduction of IL-10 levels in LdWT infection (Figure 7B, CD200R^+^ panel, *p* = 0.0065, Figure S4 in Supplementary Material), suggesting that blocking CD200 signaling could alter the CD4^+^ T cell characteristics. Similar reduction was not evident in LdCen^−/−^ infection presumably due to low baseline induction of CD200R (Figure 7B, CD200R^−^ panel; Figure S4 in Supplementary Material). Since blocking of CD200–CD200R signaling following LdWT infection correlated with the suppression of IL-10 producing CD4^+^ T cells, we were particularly interested to further characterize these cells. Our recent studies pointed that the IL-10-producing cells could be of Tr1 phenotype (39). To test whether induction of CD200R primes the CD4^+^ T cells to acquire Tr1 phenotype, a non-Treg-type cell that has been identified as a source of IL-10, we labeled the cells with Tr1-specific markers 14 days post infection [CD49b and CD223, also known as LAG3 (40)]. Results showed that indeed CD200R expression is correlated with the acquisition of LAG3 and CD49b markers on CD4^+^ T cells in LdWT infection compared to LdCen^−/−^ immunization (Figure 7C, *p* = 0.0006 between LdWT and LdCen^−/−^). Furthermore, blocking of CD200 significantly reduced the Tr1 cells in LdWT infection (Figure 7C). In contrast, CD4^+^ T cells with low CD200R expression showed overall lower expression of CD49b and LAG-3, and the difference was not significant between LdWT and LdCen^−/−^ groups (Figure 7D). Interestingly, blocking of CD200 following immunization with LdCen^−/−^ enhanced the IFNγ-producing (Figure 7E, *p* = 0.0357) and IFNγ/TNFα/IL-2-producing (Figure 7E, *p* = 0.0132) CD4^+^ T cells compared to unblocked controls.

**Figure 8:**
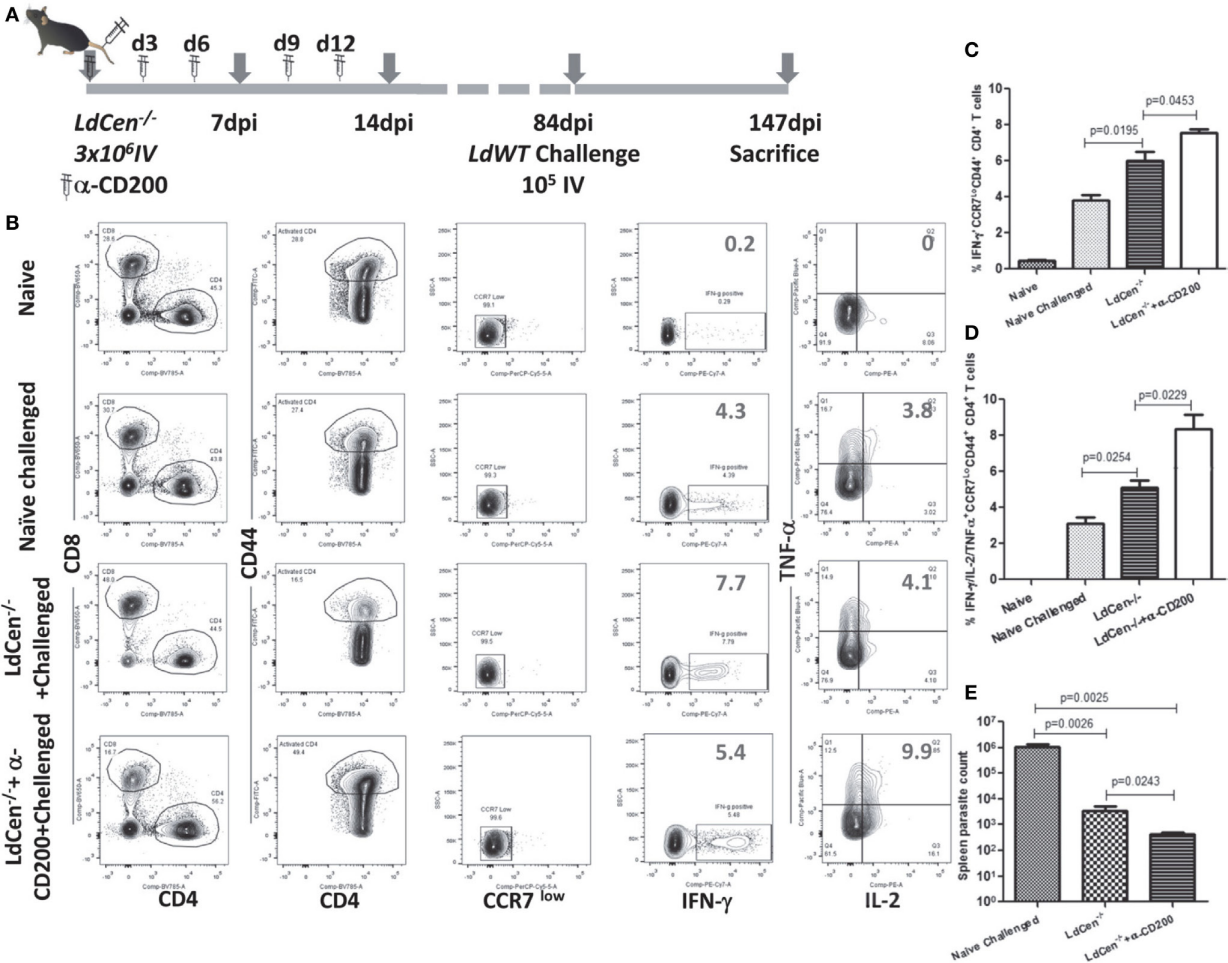
Effect of α-CD200 blocking on the function of CD4^+^CD44^+^ T cells in LdCen^−/−^-immunized mice. **(A)** Schematic showing the schedule of immunization and administration of α-CD200 antibodies. After 12 weeks of immunization, animals were challenged intravenously with virulent metacyclic *Leishmania donovani* promastigotes and sacrificed after 9 weeks. **(B)** A flow cytometry analysis showing representation of IFNγ, TNFα, and IL2 cytokine-producing CD4^+^ T cells of mice spleens that received either α-CD200 or no antibodies. **(C)** IFNγ-producing CCR7 low activated (CD44^+^) CD4^+^ T cells indicating an effector T cell response. **(D)** IFNγ, TNFα, and IL2-producing CCR7 low activated (CD44^+^) CD4^+^ T cells, indicating a multifunctional effector T cell response. **(E)** Spleen parasite load in LdCen^−/−^ immunized challenged and/or treated with α-CD200 antibodies mice. Data are obtained from experiments with 4–6 animals in each group.

To assess the impact of blocking CD200–CD200R signaling on the long-term protective immunity and on the parasite clearance, we immunized the mice with LdCen^−/−^ parasites and coadministered α-CD200 antibodies as described in Figure 8A. The mice were challenged with virulent metacyclic *L. donovani* promastigotes after 84 days of immunization (Figure 8A). To assess the post-challenge IR (147 dpi), we examined the splenic CD4^+^ T cells for the multifunctional phenotype in the immunized mice. Upon restimulation with *Leishmania* antigens *in vitro*, CD4^+^ multifunctional T cells (IFNγ/TNFα/IL-2 producing T cells) from these groups of mice were identified using flow cytometric analysis (Figure 8B). Results showed that the blocking with α-CD200 antibodies could not only enhance the IFNγ producing cells (*p* = 0.0453) (Figure 8C) but also the multifunctional CD4^+^ T cells (IFNγ, TNFα, and IL2, *p* < 0.05) in LdCen^−/−^-immunized challenged mice as compared to non-treated animals (Figure 8D).

We measured the parasite burden in the naïve challenged and immunized challenged mice to confirm if the induction of multifunctional response upon blocking with α-CD200 antibodies resulted in a corresponding reduction in parasite burden. Results showed that splenic parasite burden was significantly less in LdCen^−/−^-immunized challenged mice compared to naive challenged mice. Treatment with α-CD200 antibody further reduced the parasite burden compared to the non-treated group (Figure 8E). Furthermore, in an independent experiment, we also evaluated if CD200 blocking could control proliferation of virulent parasites in an LdWT infection. A group of naïve animals were treated with α-CD200 antibodies and infected with virulent parasites and assessed for splenic parasite load. *In vivo* blocking significantly (*p* = 0.0027) reduced parasite burden 4 weeks post infection in α-CD200 antibody-treated animals as compared to naïve infected that further confirmed the protective capabilities of CD200 blocking (Figure S5 in Supplementary Material).

**Figure 9:**
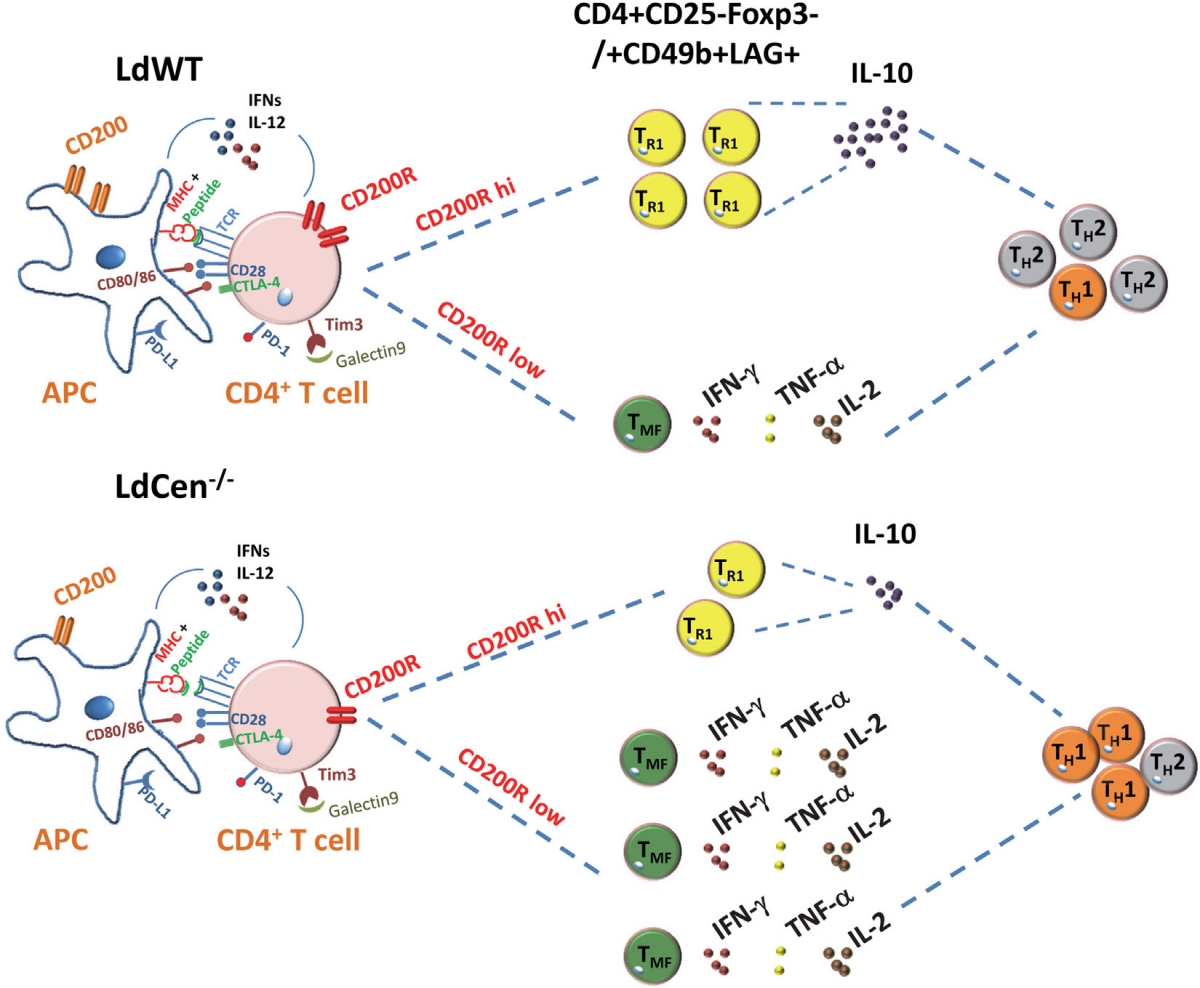
Schematic showing the role of CD200–CD200R signaling in altering the host immunity induced by *Leishmania* infection. In *Leishmania* infection, the high induction of CD200 and CD200R results in IL-10 predominant Th2-type response. The limited expression of CD200-mediated signaling by LdCen^−/−^ parasites produce more functional and robust Th1-type response indicated by the multicytokine producing CD4^+^ T cells following immunization. Other coinhibitory signaling molecules such as CTLA-4, PD-L1, TIM3, and their ligands that may impact the Th1/Th2 immunity are shown.

Taken together, our results demonstrated the role of CD200–CD200R signaling in the Th1 type of protective response induced by LdCen^−/−^ parasites. The impact of CD200–CD200R signaling on LdCen^−/−^ vaccine-induced immunity is depicted in schematic diagram in Figure 9.

## Discussion

The important role of immune stimulatory and immune inhibitory signals in shaping the adaptive immunity is increasingly being recognized. Much recent success in cancer clinical trials that blocked immune inhibitory signals, predominantly PD-L1, CTLA-4, and CD200 further underscored the importance of these inhibitory signals in reinvigorating the dysfunctional T cell responses (41). Previous studies in VL and malaria have shown the role of PD-1 and CTLA-4 in pathogenesis due to the induction of dysfunctional T cells (42, 43). Blocking of PD-1 or CTLA-4 signaling has shown clinical benefit not only in cancer and viral infections but also in *ex vivo* parasitic infection studies as well (44). However, the role of immune inhibitory molecules has not been investigated in vaccine-induced immunity against various infectious diseases including Leishmaniasis. Live attenuated *Leishmania* vaccines such as LdCen^−/−^ have shown reproducible protection in several animal models. Unlike recombinant antigen vaccine formulations that require administration of multiple doses of antigen(s) and adjuvant combination to induce a measurable response, LdCen^−/−^ parasites alone have shown remarkable degree of immunogenicity in single dose administration regimens. Therefore, it stands to reason that LdCen^−/−^ parasites might be altering immune stimulatory and inhibitory signals due to inherent attenuation of virulence as observed previously (10–13). Earlier studies with virulent *L. donovani* infection have shown high levels of c-inhibitory molecules such as PD-L1 and CTLA-4 (42, 43). However, to our knowledge, the role of coinhibitory molecules in the *Leishmania* vaccine-induced immunity has not been studied. In this study, we specifically focused in studying the role of CD200–CD200R signaling-mediated effects in the vaccine-induced immunity. Toward this end, we have investigated the expression of CD200 and CD200R on infected DCs and antigen-experienced CD4^+^ T cells, respectively, in the present study both *in vitro* and *in vivo*. Our results showed that LdCen^−/−^ infection resulted in consistently reduced CD200 expression compared to LdWT infection in BMDCs, splenic DCs *in vitro* and *in vivo*. Expression of CD200 on DCs infected with LdCen^−/−^ addback is restored to the levels as observed in LdWT infection thereby indicating that the altered expression of CD200 is tied to the attenuation of virulence in LdCen^−/−^. These results are consistent with our previous results that re-expression of centrin fully restored the virulence (11). Compared to LdWT infection, the percentage of CD200^+^ DCs and CD200R^+^ CD4 T cells was found significantly less in LdCen^−/−^-immunized animals. Furthermore, induction of CD200R expression in antigen-experienced CD4^+^ T cells was found highly correlative to the loss of ability to produce inflammatory (Th1) cytokines in LdCen^−/−^-immunized animals. Most remarkably, CD200R^+^ CD4^+^ T cells were found to produce more IL-10 than CD200R^−^ CD4^+^ T cells, which suggested that CD4^+^CD44^+^CD200R^+^ phenotypes could contribute to the production of IL-10 in leishmanial infection. The percent population of CD4^+^CD200R^−^ T cells, along with the proliferation and a potential to secrete multiple cytokines, was high in LdCen^−/−^-immunized animals. The CD200 blocking directed CD200R^+^ T cells to acquire Th1-type functional characteristics that further suggested that reduced CD200-CD200R expression may help antigen experience CD4^+^ T cells to produce inflammatory cytokines as observed in LdCen^−/−^-immunized animals. We have previously reported strong protection abilities of these parasites and herein provide one of the possible mechanisms *via* control of coinhibitory molecules such as CD200 and CD200R, resulting in CD4^+^ T cells to acquire Th1-type multi-functionality (10–14).

Leishmanial infections are also characterized by decreased cellular proliferation of CD4^+^ T cells along with increased CD4^+^IL4^+^/IL10^+^ T cells (45). We observed more IL-10^+^ CD4^+^CD200R^+^ T cells, which also showed reduced proliferative abilities in LdWT-infected mice. T cell proliferation is not only required for protective immunity and antigen clearance but also for healing and establishment of memory T cells (46). LdCen^−/−^ immunization has shown to induce proliferation of both CD4^+^ and CD8^+^ T cells, which may result in the generation of memory cells, as these parasites are known to induce long-lasting protective immunity (16, 47). In DC, T cells coculture experiment, the blockade of CD200 resulted in increased proliferation of OT-II TCR transgenic CD4^+^ T cells in LdWT and LdCen^−/−^ infections. Of the two, LdCen^−/−^ infection produced much higher proliferation than LdWT, which can be related to properties of attenuation of virulence to induce cellular proliferation that could be due to the subdued CD200 expression.

A balance between IR and IT does not only help to restore immunological sequences during a pathogenic state but also facilitate establishment of protective memory (48, 49). Furthermore, the hyperactivated state of immunity may lead to various pathological conditions such as allergy, autoimmunity, inflammation, and possible death of host (48). It is evident from the previous studies that the purpose of activation of secondary inhibitory receptors such as CD200R is to control hyperactivation that also contributes to restore the required equilibrium between IR and IT for a better protection (26). This study clearly demonstrates that rebalancing IR and IT through the alteration of CD200–CD200R signaling among various other factors could help to establish a strong protective immunity as evidenced by the effect of low CD200 on DCs and low CD200R on CD4^+^ T cells in LdCen^−/−^ immunization. Our results also show that the degree of CD200–CD200R signaling can have potentially different outcomes in terms of pathogenesis and protection as demonstrated by the multifunctional T cell response and proliferation responses upon blocking CD200 expression.

To curb leishmanial pathogenesis, Th1 cytokines such as TNFα, IFNγ, and IL-12 produced by T cells are not only necessary to establish adaptive IR but also help phagocytic cells to kill intracellular parasites (7). The protozoan parasites are known to alter host protective immunity by many ways (50, 51). *L. donovani* infection preferentially promotes the development of a Th2 phenotype to produce anti-inflammatory cytokines such as IL-4, IL-5, and IL-10 that inhibit proliferation and function of immune cells to establish pathogenesis (50, 51). In *Leishmania* infection, the IL-10 induces pathogenesis and shown to be directly involved in disease progression and control (52). So far, the cellular sources of IL-10 remain unidentified in leishmaniasis. It was believed that classical Treg cells probably produce IL-10 but studies have ruled out this in human leishmaniasis (53). This observation is of particular significance since leishmanial pathogenesis is characterized by increased production of IL-10 and compromised production of pro-inflammatory cytokines by host immune cells (8, 9). However, the phenotype of T cells which produce IL-10 is not identified in leishmanial pathogenesis. Previous studies from us and other laboratories suggested that Tr1 cells, a subset of Treg could be the population that produces IL-10 (39, 54, 55). In the present study, we have shown that Tr1 cells expressing CD200R produce IL-10 in *L. donovani* infection and are significantly reduced in LdCen^−/−^ infection. Therefore, this study further extends previous observations that CD4^+^CD44^+^CD200R^+^ T cells could be IL-10 producing phenotypes of CD4^+^ T cells in *Leishmania* infection and modulating the expression of IL-10 in such cells by α-CD200 could enhance the immunization effect by LdCen^−/−^ parasites. These findings could help to design attenuated vaccines to control the overactivation of IT mechanisms to produce more functional CD4^+^ cells and better protective immunity.

The interaction of immune inhibitory ligands and their receptors in APC and T cells, respectively, also provides feedback inhibition to activated T cells that can be reversed by blockade of their ligands (18, 44). The two inhibitory receptors on activated T cells, PD1 and CTLA4, have already been shown to promote leishmanial pathogenesis by inducing a dysfunctional state in activated T cells (41, 56). During acute and chronic conditions, the primary inhibitory response of T cells is controlled by PD1 and CTLA4 receptors as a means of limiting inflammatory cytokine production by activated T cells; however, in chronic infection their persistent activation may also lead to T cell anergy or exhaustion (18, 57, 58). A study in *L. major* suggested that increased PD1 expression on CD4^+^ and CD8^+^ T cells is responsible for exacerbated pathogenesis mainly due to impaired T cells priming and exhaustion (59, 60). The PD1/PDL1 blockade has been found to restore both CD4^+^ and CD8^+^ T cells function, especially in terms of high IFNγ and low IL-10 production, in *L. infantum* (61) and *L. major* (59) infections. However, exhausted CD8^+^ T cells, due to high PD1 and CTLA4 surface expression in *L. donovani*-infected patients do not revert to their normal function even after antigens exposure (52). These studies suggest the acquisition of PD1/CTLA4 on activated T cells silence their function rather than affecting their effector properties in leishmanial pathogenesis.

In comparison to PD1/CTLA4, the role of CD200–CD200R axis is much less studied in various pathogenic states. The augmented CD200 expression not only attenuates antigen-specific Th1 cytokines such as IFNγ and TNFα production but also direct them to acquire Th2 phenotype (expression of IL-4/IL-10 cytokines in viral and tumor pathogenesis) (6, 28, 35). CD200^−/−^ mice inoculated with influenza virus developed more severe disease in comparison to wild-type mice due to the lack of control of the initial IR, suggesting the involvement of CD200–CD200R axis in T cell functions (33). More broadly, in chronic *Salmonella enterica* and *Schistosoma mansoni* infections, the expression of CD200R on activated CD4^+^ T cells confirmed that prolonged stimulation or a chronic condition may lead to sustained upregulation of CD200R on these cells (62). This study further confirmed that moderately increased expression of CD200R on CD4^+^ T cells is associated with significant reduction in their multi-functionality in terms of IFNγ, IL-2, and TNFα and with increased IL-10 production. In contrast to the wild-type infection that results in chronic infection, LdCen^−/−^ parasites which do not persist appear to alter the inhibitory signals to yield a different outcome. The protection induced by LdCen^−/−^ parasites is mediated by the multifunctional T effector cells and subsequent generation of T cell memory response (63). We observed more percentage of multifunctional cells on CD4^+^CD44^+^CD200R^−^ T cells in both LdWT challenged and LdCen^−/−^-immunized challenged animals; however, it was more pronounced in LdCen^−/−^-immunized animals. These findings further suggested that enhanced CD200R expression in CD200R^+^ T cells in wild-type infection is responsible for loss of CD4^+^ T multi-functional Th1 response and signals them to acquire a Th2-type phenotype as evidenced by high number of IL-10 producing CD4^+^CD44^+^CD200R^+^ T cells.

Therapeutic strategies involving α-PD1, α-CTLA4, and α-CD200 antibodies have already been shown to alleviate suppressed IR and enhanced T cell-mediated immunity in various cancers (64–66). In infectious pathogenesis too, the blockade of PD1/PDL1 in both prophylactic and preventive vaccination has found to be very beneficial to control viral pathogenesis (67, 68). Our results showed that blocking CD200 increased multi-cytokine-producing CD4^+^ T cells in LdCen^−/−^-immunized animals upon virulent parasite challenge with a concomitant reduction in parasite burden. These findings further suggested that the CD200 blockade at early stage of infection with vaccine antigens may induce better protection. Furthermore, manipulation of CD200–CD200R axis may provide an alternative therapeutic strategy to boost drug response to control leishmanial pathogenesis.

In summary, this study provides a possible mechanism of protection by genetically modified live attenuated *Leishmania* vaccine through moderate reduction in the level of CD200 in DCs and CD200R in antigen-experienced CD4^+^ T cells that produce multiple cytokines, a predictor of protective immunity.

## Author Contributions

RS and SG: conceived, designed, and performed the experiments and cowrote the manuscript. NI and AK: performed animal and flow cytometry studies. MG: assisted in design of the study, statistical analysis, and manuscript writing. HN: conceived, designed, directed, and supervised the complete study.

## Conflict of Interest Statement

The authors declare that the research was conducted in the absence of any commercial or financial relationships that could be construed as a potential conflict of interest.

## Supplementary Material

Figure S1 Percentage of infected DCs, total number of parasites per 100 DCs, and NO production in LdWT and LdCen^−/−^ infection. **(A)** Bone marrow-derived DCs were differentiated in the presence of GM-CSF and IL-4 and infected with LdWT and LdCen^−/−^ parasites in a ratio of 1:10 (DC:parasite) for 6 h, after wash-infected DCs were further incubated for desired time points. No statistical difference between wild-type (LdWT) and centrin-deleted parasites (LdCen^−/−^) at all time points post infection. **(B)** At 24 and 48 h, the number of intracellular parasites (amastigotes) was significantly less in LdCen^−/−^-infected cells. **(C)** The LdCen^−/−^ infection resulted in induced NO production as compared to LdWT infection after 4 and 24 h.

Figure S2 Production of inflammatory cytokines by infected DCs. **(A–D)** The levels of IL-12 p70, TNFα, IFNγ, and IL-1β were measured at 24, 48, and 72 h post infection in culture supernatants. **(A,B)** The levels of IL-12 and TNFα were found increased in LdCen^−/−^-infected cells 24 h post infection. After 48 h post infection, a sharp decline in their levels was observed in both infections that was insignificant between groups. **(C,D)** The difference in the level of IFNγ and IL-1β was also insignificant but at 72 h the level of IL-1β was significantly high in LdCen^−/−^-infected cells.

Figure S3 Parasite burden in the spleen of LdWT and LdCen^−/−^-infected animals. In LdWT-infected animals, the parasite burden as determined by serial dilution was significantly more at both days 7 and 14 post infection as compared to LdCen^−/−^-immunized animals.

Figure S4 Evaluation of IL-10 producing CD4^+^ T cells in CD200R^−^ and CD200R^+^ groups. IL-10 producing CD200R^−^ and CD200R^+^ T cell populations 14 days post infection are shown. The α-CD200 antibody treatment was done as shown in Figure 7A.

Figure S5 Evaluation of CD200 blocking on the proliferation of virulent LdWT parasites in independent experiments in mice. A group of naïve animals were treated with α-CD200 antibodies and infected with virulent LdWT parasites and assessed for splenic parasite load. *In vivo* blocking with α-CD200 antibodies significantly reduced parasite burden 4 weeks post infection in treated animals as compared to naïve infected animals. Data are obtained from experiments with six animals in each group.
