# Supplementary figures and images for: Centrin-Deleted Leishmania donovani Parasites Help CD4+ T Cells to Acquire Th1 Phenotype and Multi-Functionality Through Downregulation of CD200–CD200R Immune Inhibitory Axis
Source: Front Immunol. 2018 Jun 4;9:1176. doi: 10.3389/fimmu.2018.01176 (PMC5994488; doi:10.3389/fimmu.2018.01176)

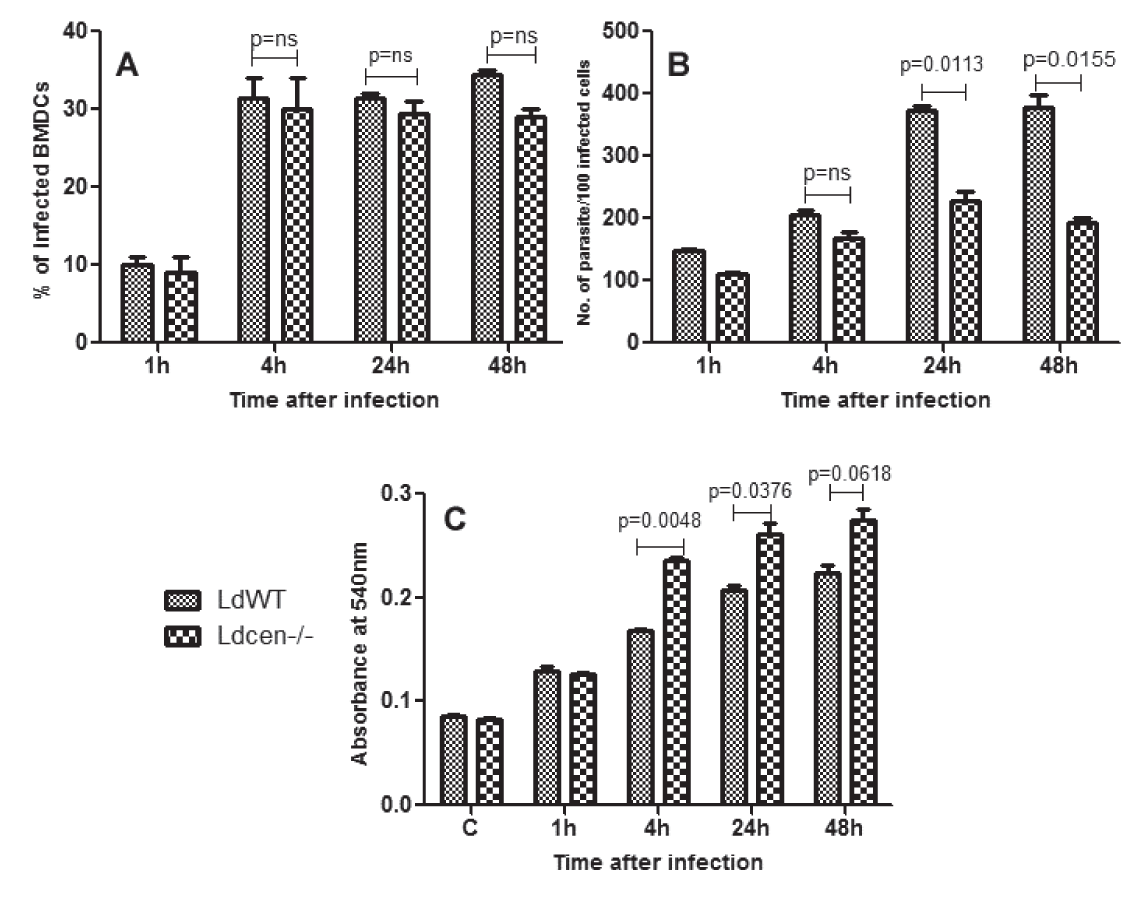

Supplement: Figure S1 — Percentage of infected DCs, total number of parasites per 100 DCs, and NO production in LdWT and LdCen−/− infection. (A) Bone marrow-derived DCs were differentiated in the presence of GM-CSF and IL-4 and infected with LdWT and LdCen−/− parasites in a ratio of 1:10 (DC:parasite) for 6 h, after wash-infected DCs were further incubated for desired time points. No statistical difference between wild-type (LdWT) and centrin-deleted parasites (LdCen−/−) at all time points post infection. (B) At 24 and 48 h, the number of intracellular parasites (amastigotes) was significantly less in LdCen−/−-infected cells. (C) The LdCen−/− infection resulted in induced NO production as compared to LdWT infection after 4 and 24 h. [file image_1.TIF]

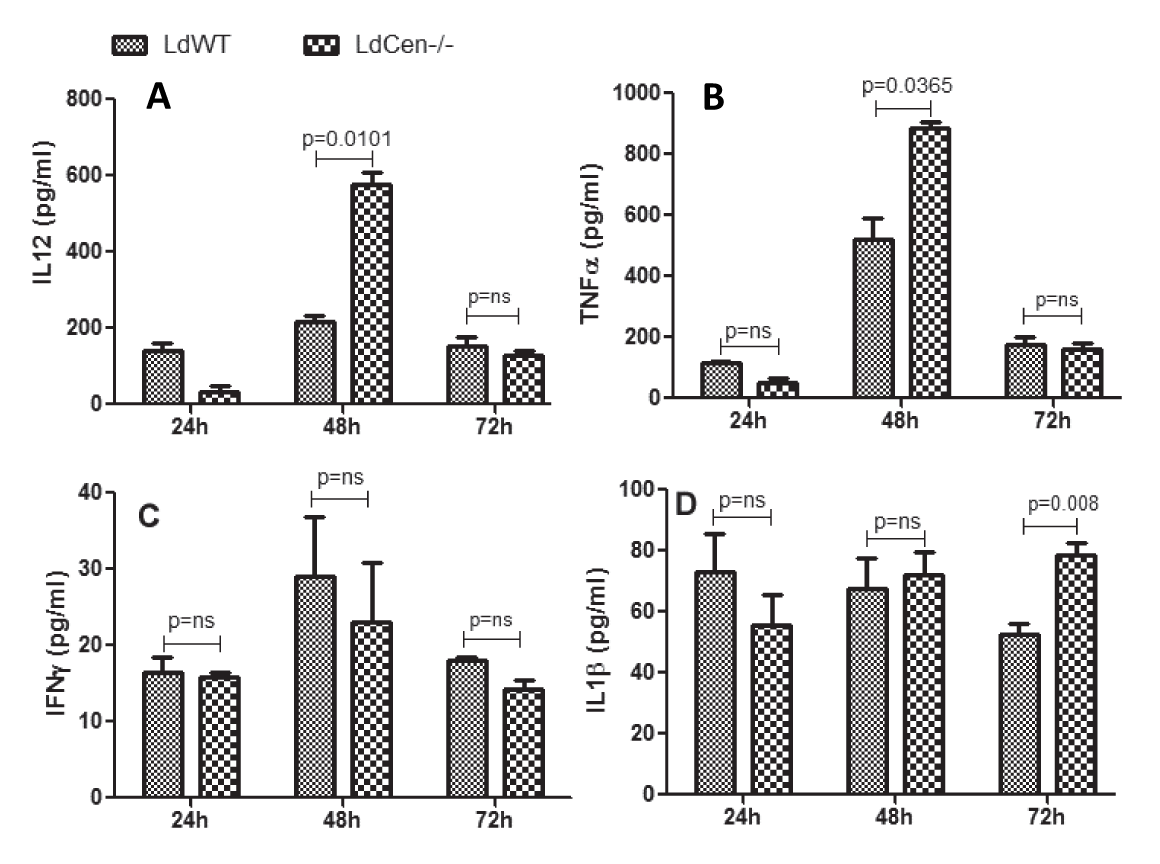

Supplement: Figure S2 — Production of inflammatory cytokines by infected DCs. (A–D) The levels of IL-12 p70, TNFα, IFNγ, and IL-1β were measured at 24, 48, and 72 h post infection in culture supernatants. (A,B) The levels of IL-12 and TNFα were found increased in LdCen−/−-infected cells 24 h post infection. After 48 h post infection, a sharp decline in their levels was observed in both infections that was insignificant between groups. (C,D) The difference in the level of IFNγ and IL-1β was also insignificant but at 72 h the level of IL-1β was significantly high in LdCen−/−-infected cells. [file image_2.TIF]

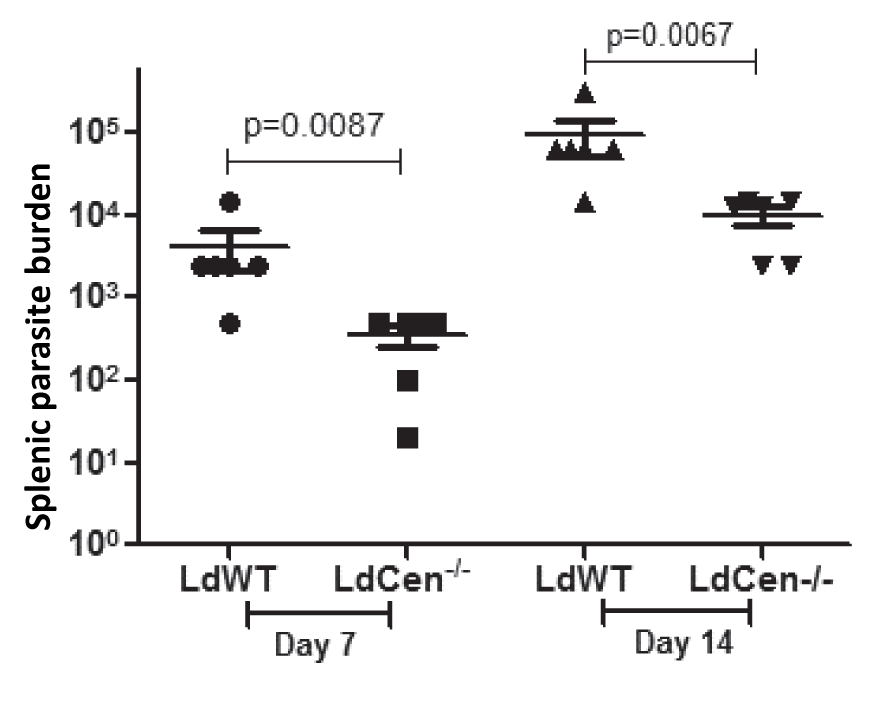

Supplement: Figure S3 — Parasite burden in the spleen of LdWT and LdCen−/−-infected animals. In LdWT-infected animals, the parasite burden as determined by serial dilution was significantly more at both days 7 and 14 post infection as compared to LdCen−/−-immunized animals. [file image_3.TIF]

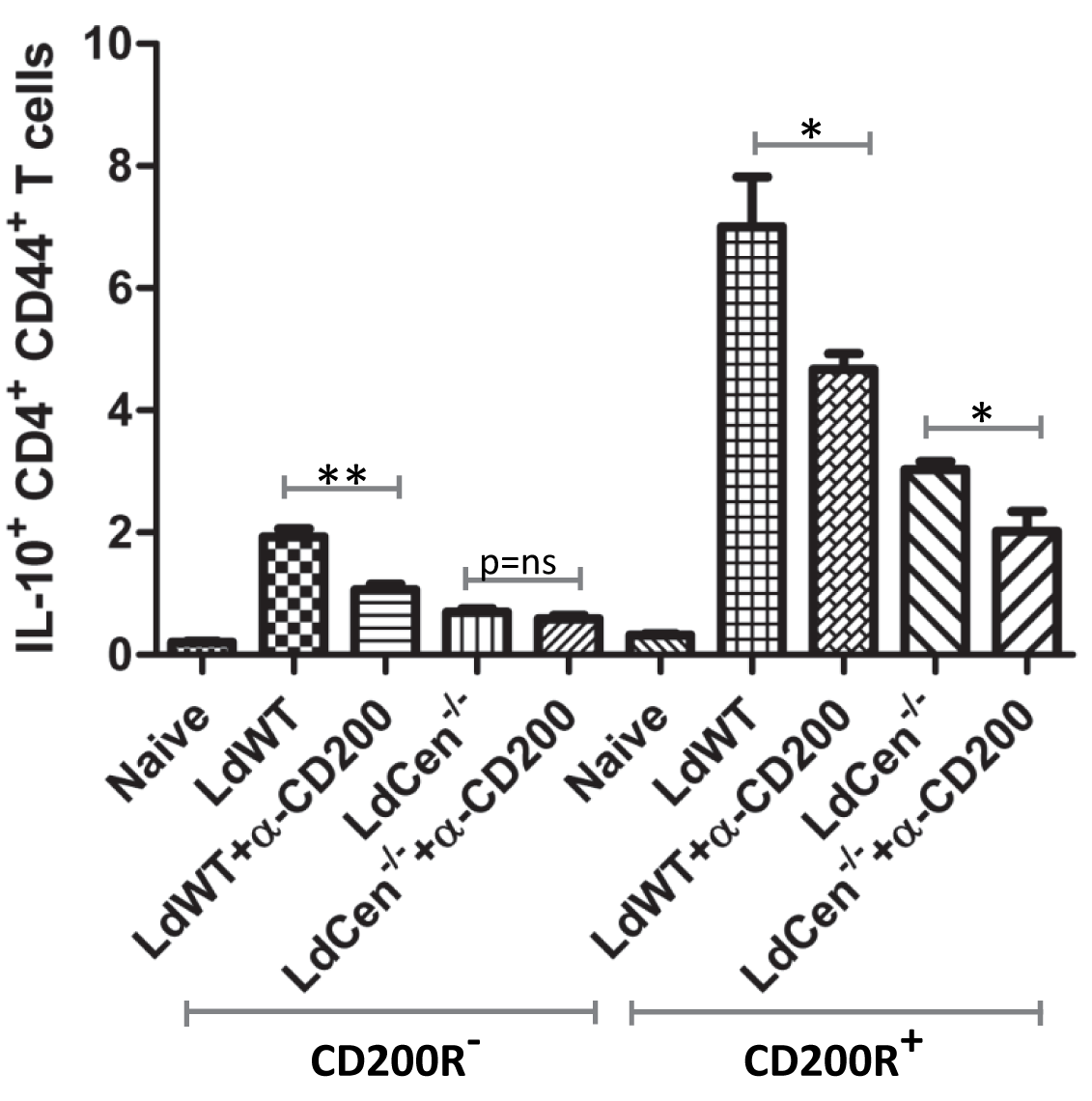

Supplement: Figure S4 — Evaluation of IL-10 producing CD4+ T cells in CD200R− and CD200R+ groups. IL-10 producing CD200R− and CD200R+ T cell populations 14 days post infection are shown. The α-CD200 antibody treatment was done as shown in Figure 7A. [file image_4.TIF]

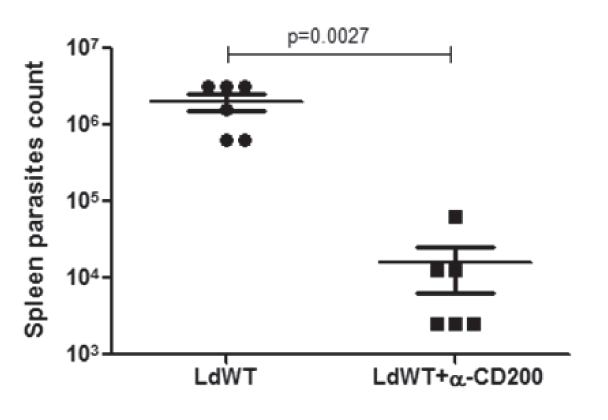

Supplement: Figure S5 — Evaluation of CD200 blocking on the proliferation of virulent LdWT parasites in independent experiments in mice. A group of naïve animals were treated with α-CD200 antibodies and infected with virulent LdWT parasites and assessed for splenic parasite load. In vivo blocking with α-CD200 antibodies significantly reduced parasite burden 4 weeks post infection in treated animals as compared to naïve infected animals. Data are obtained from experiments with six animals in each group. [file image_5.TIF]
